# Lentiviral vector mediated gene therapy for type I Dent disease ameliorates Dent disease-like phenotypes for three months in ClC-5 null mice

**DOI:** 10.1016/j.omtm.2022.09.009

**Published:** 2022-09-24

**Authors:** Manish Kumar Yadav, Kyung Whan Yoo, Anthony Atala, Baisong Lu

**Affiliations:** 1 Wake Forest Institute for Regenerative Medicine, Wake Forest University School of Medicine, 391 Technology Way, Winston-Salem, NC, USA

**Keywords:** CLCN5, megalin, CRISPR-Cas9, gene therapy, lentiviral vector, immune response, Dent disease, mouse model

## Abstract

Type 1 Dent disease is caused by changes in chloride voltage-gated channel 5 (*CLCN5*) gene on chromosome X, which causes the lack or dysfunction of chloride channel ClC-5. Affected subjects show proteinuria and hypercalciuria, and eventually develop end-stage kidney disease. Currently there is no cure for this disease. Here, we used CRISPR-Cas9 technology to develop a *Clcn5* mouse model with 95% of the ClC-5 coding region deleted. These mutant mice showed obvious Dent disease-like phenotypes. We used lentiviral vectors to deliver human *CLCN5* cDNA into the kidneys of mutant mice by retrograde ureter injection and observed increased megalin expression, improved diuresis, and decreased urinary calcium and protein excretion, which persisted for 3 months. The therapeutic effects diminished 4 months after gene therapy. Our data suggest that immune responses to the transgene products most likely explain the loss of gene therapy effects. This study suggests that gene therapy could be a promising approach to treat Dent disease, but more work is needed to achieve sustained therapeutic effects.

## Graphical abstract

**Figure:**
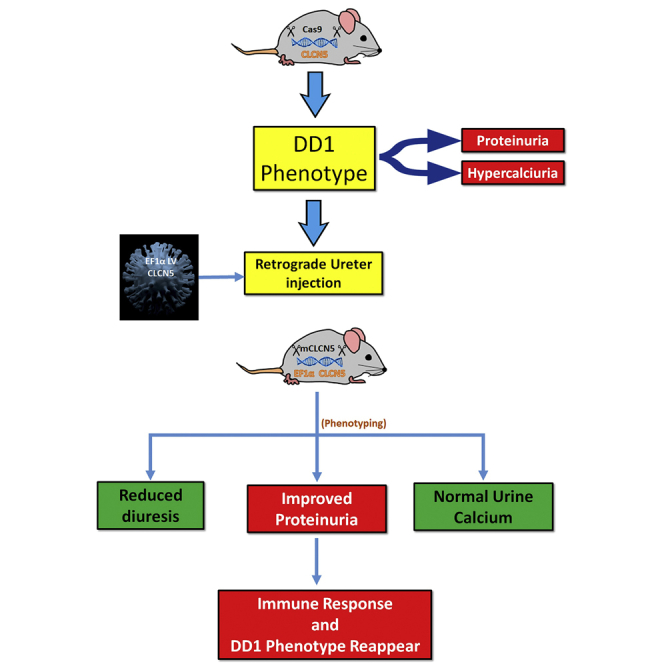

## Teaser

Type 1 Dent disease is one of the kidney tubule disorders caused by hereditary mutations without cures. Here, we show that local gene therapy is effective for improving phenotypes in Dent disease model mice. Our work suggests that gene therapy may be a promising option for treating kidney tubule disorders.

## Introduction

Dent disease (DD) is a chronic kidney disorder affecting mainly males. The proximal tubules of affected individuals cannot reabsorb small-molecular-weight proteins, water, and other materials that have been filtered from the bloodstream, resulting in an abnormally large amount of proteins in the urine, along with excess urinary calcium (hypercalciuria), calcium deposits in the kidneys (nephrocalcinosis), and kidney stones (nephrolithiasis).^1^ Between 30% and 80% of affected males develop end-stage renal disease between the ages of 30 and 50 years.^2^

About 60% of DD cases are caused by changes in the *CLCN5* gene (Gene ID: 1184)^3^; this type of DD is called type I DD (DD1, MIM: 300009). About 15% of DD cases are caused by changes in the *OCRL* gene^4^ (type II DD). Both DD-causing genes are X-linked. *CLCN5* (OMIM: 300008) encodes a 746 amino acid electrogenic Cl–/H+ exchanger (ClC-5), which plays an important role in receptor-mediated endocytosis in proximal tubule epithelial cells.^5^, ^6^, ^7^, ^8^, ^9^ Over 200 different types of *CLCN5* variants have been found in subjects with type 1 DD, and disease-causing variants are scattered throughout the coding region of the gene.^10^^,^^11^ Frameshifts, splicing, and nonsense mutations account for 29.1%, 12.4%, and 17.5% of all DD1-causing variants.^10^^,^^11^ Thus, over half of those with DD1 express little or no full-length ClC-5 protein due to nonsense-mediated mRNA decay.^12^ For these subjects, augmenting the function of the endogenous protein is not an option and restoring the expression of functional ClC-5 is necessary.

Currently there are no curative therapies for DD1. Thiazide diuretics can decrease urinary calcium excretion in males with DD1, but side effects are common.^2^ Angiotensin-converting enzyme inhibitor and angiotensin receptor blocker therapies have been tried to ameliorate proteinuria,^13^ but do not target the molecular etiology of DD1 and cannot stop its progression. A therapy that targets the molecular etiology is needed for better therapeutic effects.

In DD1, the lack of dominant variants and the small affected numbers for each type of variant make gene supplementary therapy a better choice than genome editing for developing gene therapy. Currently, adeno-associated virus (AAV) vectors are the first choice for *in vivo* systemic gene delivery.^14^ However, renal tubular cells, the major defective cells in DD1,^5^, ^6^, ^7^, ^8^, ^9^ have limited lifespans and are continuously replenished by cell division from progenitor cells or differentiated tubule cells.^15^, ^16^, ^17^ Thus, AAV vectors cannot mediate sustained target gene expression in kidney proximal tubular cells. Lentiviral vectors (LVs) may be a better choice because: (1) local gene delivery can be used to correct defects of kidney proximal tubules in DD1 subjects, and LVs can be efficiently delivered to the kidney proximal tubules by retrograde ureteral injection^18^, ^19^, ^20^, ^21^, ^22^, ^23^; (2) LVs integrate into the host cell genome and mediate long-term target expression; (3) LVs are FDA-approved as a delivery vehicle for generating CAR-T cells (KYMRIAH) for clinical use, and their safety has been demonstrated by numerous clinical trials^24^^,^^25^; and (4) LVs have larger capacity than AAVs and are compatible with possible kidney tubule cell-specific promoters to achieve tissue-specific expression.

*Clcn5* knockout mice^26^^,^^27^ and *Clcn5* p.Glu211Ala point mutation mice^7^ show phenotypes similar to those of patients with DD1. These models could be very useful for testing the effects of gene therapy, but live mice of these mutant strains are currently unavailable. Since the chloride channel (CIC) family proteins function as homodimers,^28^^,^^29^ we decided to first perform supplementary gene therapy in *Clcn5* knockout mice to avoid possible interference of endogenous malfunctioning ClC-5 protein on exogenous functional ClC-5 protein. Here, we report the generation of a ClC-5 null model by CRISPR-Cas9-mediated gene mutation, followed by LV-mediated DD1 gene therapy in ClC-5 null mice.

## Results

### CRISPR-Cas9-generated ClC-5 null mice showed DD1 typical phenotypes

**Figure 1:**
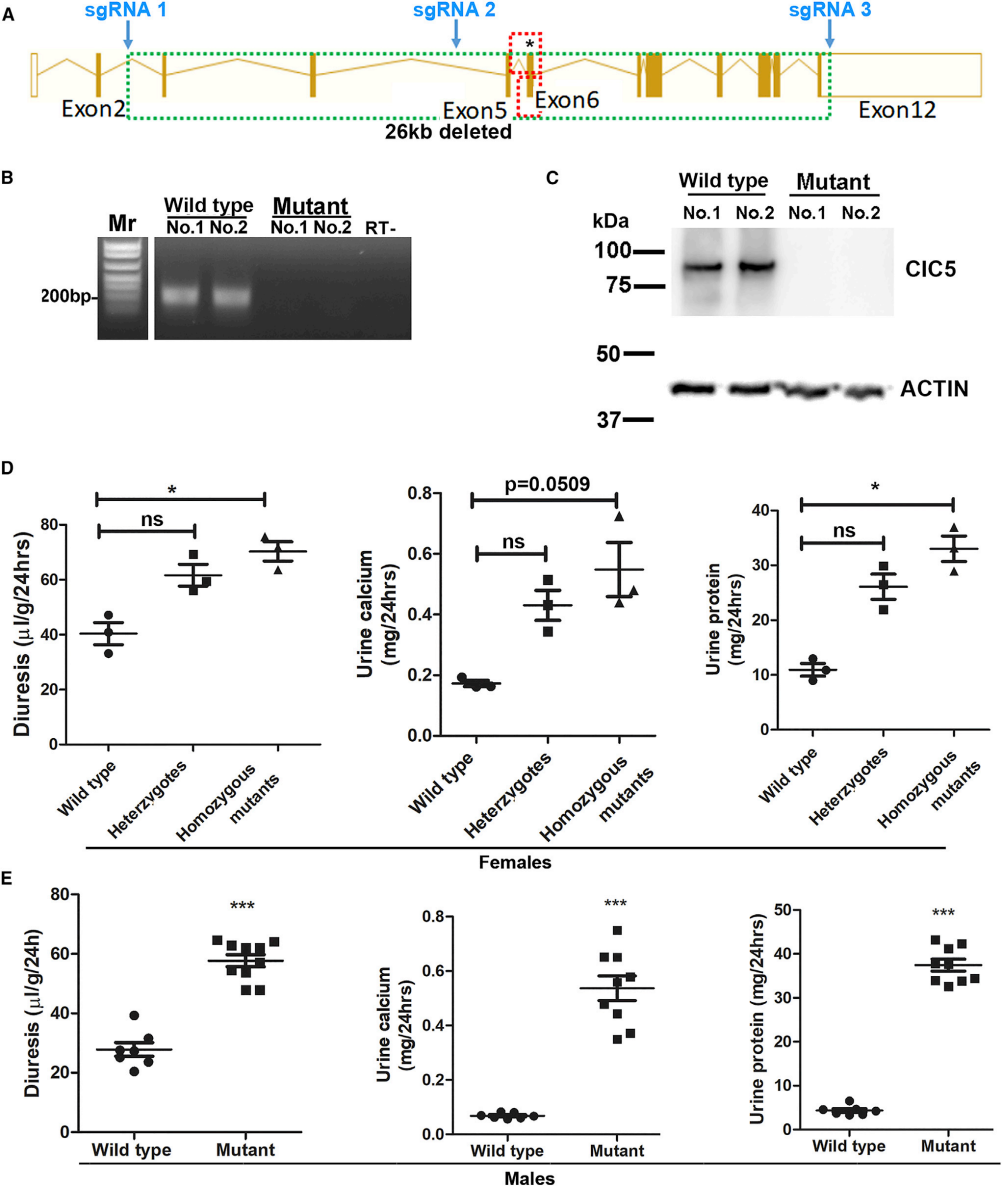
Generation and characterization of *Clcn5* knockout mice (A) Gene structure of mouse *Clcn5* and the sgRNAs used for deleting the 26 kbp region. The two dashed red boxes indicate the replaced regions in the two published gene knockout strains (Piwon and co-workers^26^^,^^27^). The asterisk indicates the position of the point mutation in the published point muation mouse model (Novarino et al.^7^). (B) RT-PCR confirming the lack of *Clcn5* mRNA expression in the kidneys of mutant mice. Two wild-type and two mutant mice (2 months old) were analyzed. “RT-”: RNA from the kidney of a wild-type mouse was directly used as a PCR template without reverse transcription. Primers were specific to mouse *Clcn5* cDNA. (C) Western blotting confirming the lack of ClC-5 protein in the kidney tissues of mutant mice. (D) Volume, calcium, and protein in urine of female mice. Wild-type, heterozygous, and homozygous mutant mice were 81 days old. ∗p < 0.05 between the indicated groups (Dunn's multiple comparison test following Kruskal-Wallis test). (E) Volume, calcium, and protein in urine of male mice. Urine samples were collected from 2- to 2.5-month-old mice. ∗∗∗p < 0.0001 between wild-type and mutant mice (Mann-Whitney test).

We used CRISPR-Cas9 to knock out the mouse *Clcn5* gene. Three guide RNAs were designed to target introns 2 and 4 and exon 12 on the gene (Figure 1A) to delete 95% of the protein coding region. The three single-guide RNAs (sgRNAs) and Cas9 mRNA were injected into fertilized mouse eggs to delete the *Clcn5* gene.

Three heterozygous female mice were obtained, each with a 26-kbp deletion in the *Clcn5* gene (Figure S1), which removed 95% of the *Clcn5* coding region. There were no other known coding genes or non-coding genes within 80 kbp around the deleted region. Progeny from one female carrier (no. 34) were used for subsequent studies.

Because the mice were generated by CRISPR-Cas9-mediated gene mutation, we analyzed possible off-targets of the three sgRNAs used (we used three rather than two sgRNAs to increase the chance of deleting the whole gene). Predicted off-targets with no more than one mismatch in the seed region (which greatly inhibits Cas9 cleavage) had at least a 3 nt mismatch with the sgRNAs (Tables S1–S3). Only one off-target (for sgRNA 2) hit the exon of a protein coding gene (*Itgb6*). DNA of this region was amplified from a male mutant mouse and sequenced. No mutations or heterozygosity were observed (Figure S2). Eighteen predicted off-targets fell in introns and 23 in intergenic regions. We amplified the regions of all four predicted off-targets on the X chromosome from a male mutant mouse, and detected no mutations or deletions (Figure S2). Since the off-targets on X-chromosome link with the *CLCN5* deletion and male mice have only one copy of X chromosome, successful amplification of the region also ruled out the possibility of large deletions. For off-targets on autosomal chromosomes, we sequenced the regions of all five off-targets with 3 nt mismatches to the sgRNAs, and two off-targets with 4 nt mismatches to the sgRNA (at least three off-targets were analyzed for each sgRNA). We found no mutations or heterozygosity in any of these regions in mutant mice (Figure S2).

The amino acid sequence of ClC-5 is 77% identical to those of CLCN3 and CLCN4. We predicted possible off-targets of the three sgRNAs in the mouse genome with the CRISPOR program^30^ (Tables S4–S6). None of the predicted off-targets hit those two genes. *Clcn3* and *Clcn4* expression in *Clcn5* mutant mice was normal compared with wild-type mice (see Tables S7 and S8 for qPCR MIQE reports). Altogether, the data show that the likelihood of unintentionally mutating homologous genes or any other genes was low.

We confirmed the loss of *Clcn5* expression in mutant mice by RT-PCR (Figure 1B) and western blotting (Figure 1C). The mutant mice were then investigated to compare their phenotypes to those in DD1 patients. We collected urine from wild-type and mutant mice over 24 h. Female and male mutant mice showed increased diuresis, hypercalciuria, and proteinuria (Figures 1D–1E). Wild-type female mice had higher urine calcium levels than wild-type male mice, consistent with previous studies.^31^ Heterozygous female mice showed intermediate values between wild-type and homozygous mutant female mice (although no statistic difference was observed compared with wild-type mice due to the small group numbers), suggesting haploinsufficiency. This also is consistent with reports that some human female heterozygous carriers show mild DD1 symptoms.^32^ Phenotypes in null mutant mice (6- to 7-fold increase of urinary protein and calcium) were much more severe than previously reported,^7^^,^^26^^,^^27^ possibly due to the deletion of most of the *Clcn5* coding sequence. Urine creatinine concentrations of mutant mice were similar to those of wild-type mice, suggesting that creatinine filtration in mutant mice was not greatly affected. Since *Clcn5* mutant mice only show significantly decreased glomerular filtration rates after 6 months^33^ and most of our experiments were done in younger mice, we did not further examine creatinine filtration in this study.

Fewer than 50% of male mutants in a C57/BL6 background survived (Figure S3), suggesting embryonic or perinatal loss. This partial lethality was not observed in *Clcn5* mutant mice after replacing exons 5 and 6,^26^^,^^27^ or changing Glu^211^ to Ala.^7^ Although we cannot totally rule out the possibility of unintendedly mutating other genes essential for survival, suboptimal survival in pups from all three founder females argues against this possibility. Our mutant mice were in a pure C57/BL6 background and 95% of the *Clcn5* coding region was deleted, whereas the previously reported *Clcn5* mutant mice had a mixed background and had only a small part of the gene replaced. We previously observed that *Mex3c* knockout mice also showed suboptimal survival in C57/BL6 background but not in a mixed background.^34^ The deletion of 95% of the coding region might exclude the possibility of generating partially functional ClC-5 protein. When C57/BL6 female carriers were mated with wild-type FVB/NJ males, the expected ratio of *Clcn5* mutant male mice were obtained. Thus, all mutant male mice used in the study were obtained by this breeding strategy, and female mutants were obtained by breeding female carriers and male mutants of 50% C57BL/6 and 50% FVB backgrounds.

**Figure 2:**
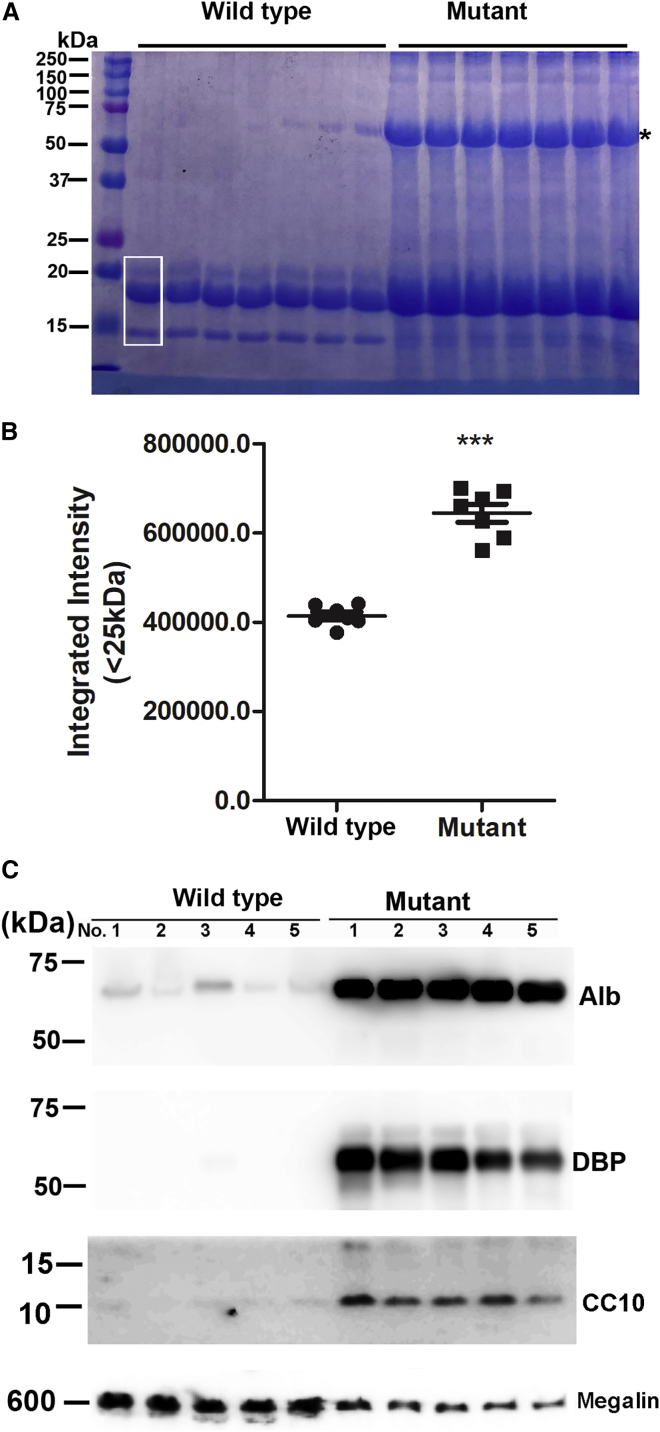
SDS-PAGE and western blotting confirmed proteinuria in *Clcn5* mutant mice (A) SDS-PAGE analysis of urine proteins of wild-type and mutant males. ∗ indicates albumin. The white box indicates the area in each lane analyzed for integrated density. (B) Integrated density of areas with proteins smaller than 25 kDa. The boxed area of each lane in (A) was analyzed by ImageJ software. ∗∗∗p < 0.001 (Mann-Whitney test). (C) Western blotting analyses of urine protein from wild-type and mutant mice. Alb, albumin; DBP, vitamin D binding protein; CC10, club cell secretory protein. For (A) and (C), equal volumes of urine samples were analyzed and each lane contained a urine sample from a different mouse.

Consistent with increased total urine protein content in mutant mice determined by BCA assays, SDS-PAGE analyses of urine protein confirmed increased small-molecular-weight proteins in urine of mutant mice (Figure 2). In addition, a very intense protein band of larger than 60 kDa was observed in urine samples of mutant mice but not in urine samples of wild-type mice (marked by ∗ in Figure 2A). Protein mass spectrometry identified 1,627 of the total 2,207 spectral matches to albumin. The second and third most frequently matched proteins were vitamin D binding protein (DBP) (71 matches) and beta-2-glycoprotein 1 (50 matches), both smaller than 60 kDa. The large number of matches to albumin and the size of the protein on SDS-PAGE suggest that this protein was albumin. We further quantified the overall protein density of bands smaller than 25 kDa (the boxed area of each lane in Figure 2A) and confirmed that the average integrated density of mutant samples was significantly higher than that of wild-type samples (Figure 2B, p = 0.0006, Mann-Whitney test). Consistently, western blotting confirmed increased levels of urine albumin, DBP, and club cell secretory protein (CC16, also called CC10) in samples from mutant mice (Figure 2C).

Due to the features of this model, it was difficult to find a protein equally presented in urine of wild-type and mutant mice as a loading control. Based on a previous report,^35^ western blotting analyses confirmed that megalin was decreased in the urine samples of mutant mice. Although megalin protein differed between wild-type and mutant mice, it was consistent with our loading equal volumes of urine for each sample. Considering the increased urine volume excreted by mutant mice, the degree of urine protein increase was larger than appeared in SDS-PAGE and western blotting analyses. Overall, the data show that we successfully knocked out the mouse *Clcn5* gene and that mutant mice showed more severe DD1 phenotypes than observed in other models.^7^^,^^26^^,^^27^

### Local kidney delivery of *CLCN5* LV restored ClC-5 expression in the kidneys of mutant mice

**Figure 3:**
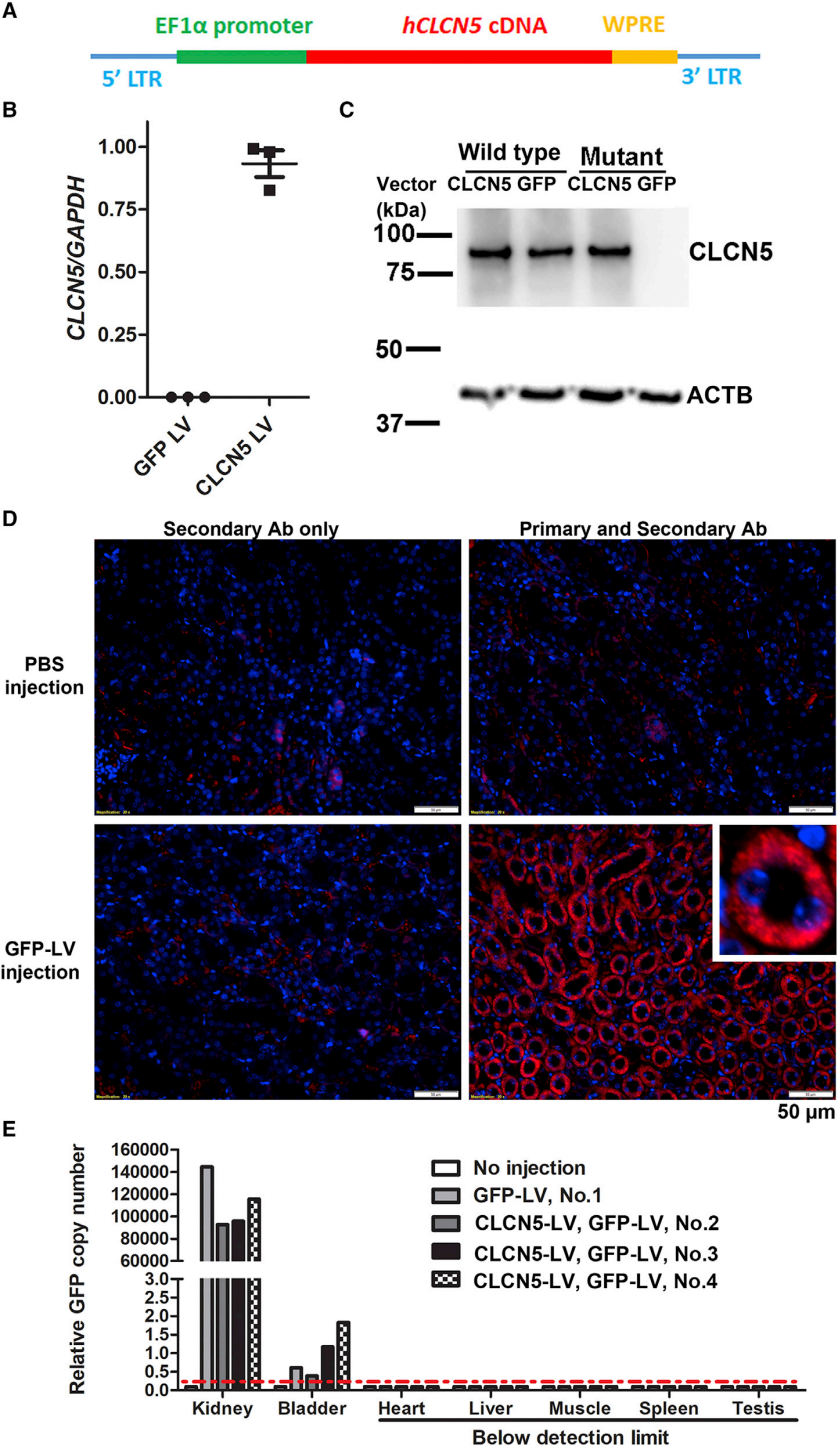
Delivery of LV to mouse kidney by retrograde ureter injection (A) Components of the human ClC-5-expressing LV. Human *CLCN5* cDNA was codon optimized to distinguish the transgene with the endogenous human cDNA. LTR, long terminal repeats. (B) LV-mediated *CLCN5* mRNA expression in HEK293T cells. ClC-5- and GFP-expressing LVs (10 ng p24) were transduced into 2.5 × 10^4^ HEK293T cells. Forty-eight hours after transduction, *CLCN5* expression was detected by qRT-PCR with primers specific for the codon-optimized human *CLCN5* mRNA (hCLCN5-F and hCLCN5-R; see Table S14 for sequences). (C) Western blots for ClC-5 protein in transduced kidney proximal tubule cells. ClC-5-expressing LVs (28 ng p24) were transduced into 2.5 × 10^5^ kidney proximal tubule cells isolated from wild-type and mutant mice. Confirmatory western blotting was performed 72 h after transduction. (D) GFP protein expression in mouse kidney 2 weeks after GFP LV delivery by retrograde ureter injection. The 6-month-old, wild-type mouse received GFP LV injection in both kidneys. GFP expression was detected by immunofluorescence (shown in red). Inset: enlarged view of a GFP-positive tubule. Nuclei were stained by 4',6-diamidino-2-phenylindole (DAPI) (shown in blue). (E) GFP LV DNA in the organs shown, as detected by qPCR 2 weeks after GFP LV delivery. Genomic DNA samples isolated from different organs were used as templates in qPCR to detect GFP DNA. Mouse no. 1 was the same mouse shown in (D). Mice nos. 2, 3, and 4 were male *Clcn5* mutant mice that received GFP LV injection 10 months following *CLCN5* LV injection. All mice were euthanized 2 weeks after GFP LV injection. The red dashed line indicates detection limit.

We developed a LV to express human *CLCN5* cDNA for possible clinical translation. Human *CLCN5* cDNA was used for two reasons: (1) human ClC-5 protein shows 97% sequence identity to its mouse homolog, and we reasoned that human ClC-5 should be functional in mouse; (2) using human *CLCN5* cDNA avoids replicated animal studies for subsequent translation into clinical use. Codon optimization was used to distinguish the transgene-expressed *CLCN5* mRNA from the endogenous *CLCN5* mRNA in human cells. The transfer plasmid was a third-generation lentiviral expression vector containing the codon optimized human *CLCN5* cDNA following the human EF1 alpha promoter (Figure 3A). A ubiquitously active promoter was used to test whether supplementing functional *CLCN5* cDNA to the kidney can ameliorate DD1 symptoms. A woodchuck hepatitis virus posttranscriptional regulatory element was included following the *CLCN5* cDNA to increase target gene expression. ClC-5*-*expressing LVs were produced with the third-generation packaging system and exogenous C*LCN*5 mRNA expression was successfully detected from HEK293T cells transduced with the LVs (Figure 3B; see Table S9 for a qPCR MIQE report).

To detect ClC-5 protein expression from the transgene, we isolated kidney proximal tubule cells from wild-type and *Clcn5* knockout mice and transduced *CLCN5* LVs into the cells. We detected ClC-5 protein in *CLCN5* LV-transduced mutant cells but not in GFP LV-transduced mutant cells (Figure 3C). Delivering *CLCN5* LV into cells from wild-type mice did not increase ClC-5 expression, suggesting that *CLCN5* is regulated in a post-transcription and/or post-translation manner. Since the main purpose of isolating these primary cells was to test ClC-5 expression from our LVs, we did not examine the possibility of contamination by other cell types, since they would not affect our ability to examine vector ClC-5 expression. The data show that the vectors mediated ClC-5 expression in kidney cells.

We tested delivering GFP LV into mouse kidney tubules (one male 6-month-old wild-type and three male 17-month-old mutants) using retrograde ureter injection. Two weeks following delivery of 100 μL GFP LV vectors (∼250 ng p24) to each kidney, immunofluorescence detected strong GFP expression in over 70% of tubule structures of all four mice (Figure 3D). We also observed GFP expression in the glomeruli (Figure S4), suggesting that this delivery method could reach the glomeruli. We collected the kidney, bladder, liver, heart, skeletal muscle, spleen, and testis of the four mice and extracted genomic DNA to detect LV DNA. We detected relatively low levels of vector DNA in the bladder, 10^5^-fold higher levels of vector DNA in the kidney, and undetectable vector DNA in all other organs (Figure 3E; see Table S10 for qPCR MIQE report). These data show that retrograde ureter injection was efficient for local tubule delivery and the chance of delivering the vectors to other organs (except for the bladder and possibly other tissues of the urinary tract) was low.

**Figure 4:**
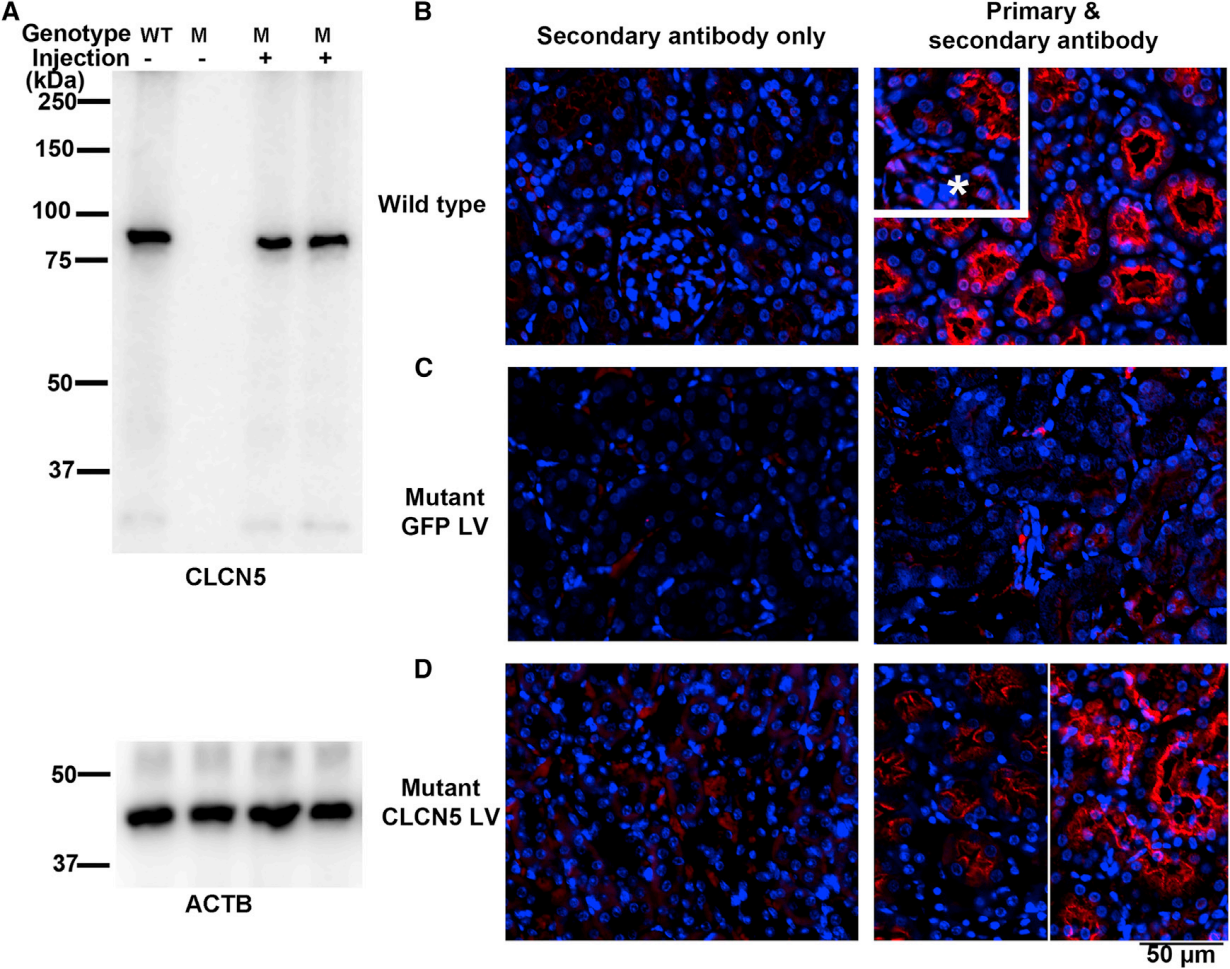
*CLCN5* LV restored ClC-5 expression in the kidneys of mutant mice (A) Western blots showing detection of ClC-5 protein in kidney tissues. Both kidneys of a 3-month-old mouse were injected with 280 ng *CLCN5* LV. The mouse was euthanized 2 weeks following LV injection. The two injected kidneys were analyzed separately. (B) Detecting ClC-5 protein by immunofluorescence in wild-type kidney. The insert shows the relatively weak ClC-5 expression in the glomeruli (marked by an asterisk). (C) Undetectable ClC-5 protein in the kidney of mutant mice without *CLCN5* LV injection. (D) ClC-5 protein in the kidneys of mutant mice 2 weeks following *CLCN5* LV injection. Lower right image: the two half images were from two injected kidneys with different ClC-5 expression levels.

We then used this method to deliver 280 ng p24 of *CLCN5* LV into the kidneys of male mutant mice. Western blotting analysis of protein extracted from kidney tissues detected ClC-5 protein in the injected kidneys but not from the non-injected kidneys 2 weeks after vector delivery (Figure 4A). Immunofluorescence analysis was performed to examine the cell types expressing transgenic ClC-5. In the kidneys of wild-type mice, ClC-5 was highly expressed in the proximal tubular epithelium (Figure 4B) but only weakly expressed in the glomeruli (Figure 4B inset, marked by ∗). Without *CLCN5* LV vector delivery, no ClC-5 expression was detected in the kidney tubules of mutant mice (Figure 4C). Two weeks following *CLCN5* LV delivery, ClC-5 was detected in kidney tubules (Figure 4D) and the glomeruli (Figure S5) of mutant mice. The ClC-5 signal in the glomeruli of *CLCN5* LV-injected mutant mice was much stronger than in the glomeruli of wild-type mice, consistent with the ubiquitous expression of the promoter used for driving *CLCN5* expression. In both wild-type and *CLCN5* LV-injected mutant mice, the strongest ClC-5 signals were detected in the apical regions of the tubular cells. This localization of exogenous ClC-5 protein showed that the LV-expressed ClC-5 protein was correctly trafficked. The data show that retrograde ureter injection could deliver LV vectors into the kidney and result in ClC-5 expression from those vectors.

### Delivering human *CLCN5* LVs to the kidneys of mutant mice ameliorated DD1 phenotypes

**Figure 5:**
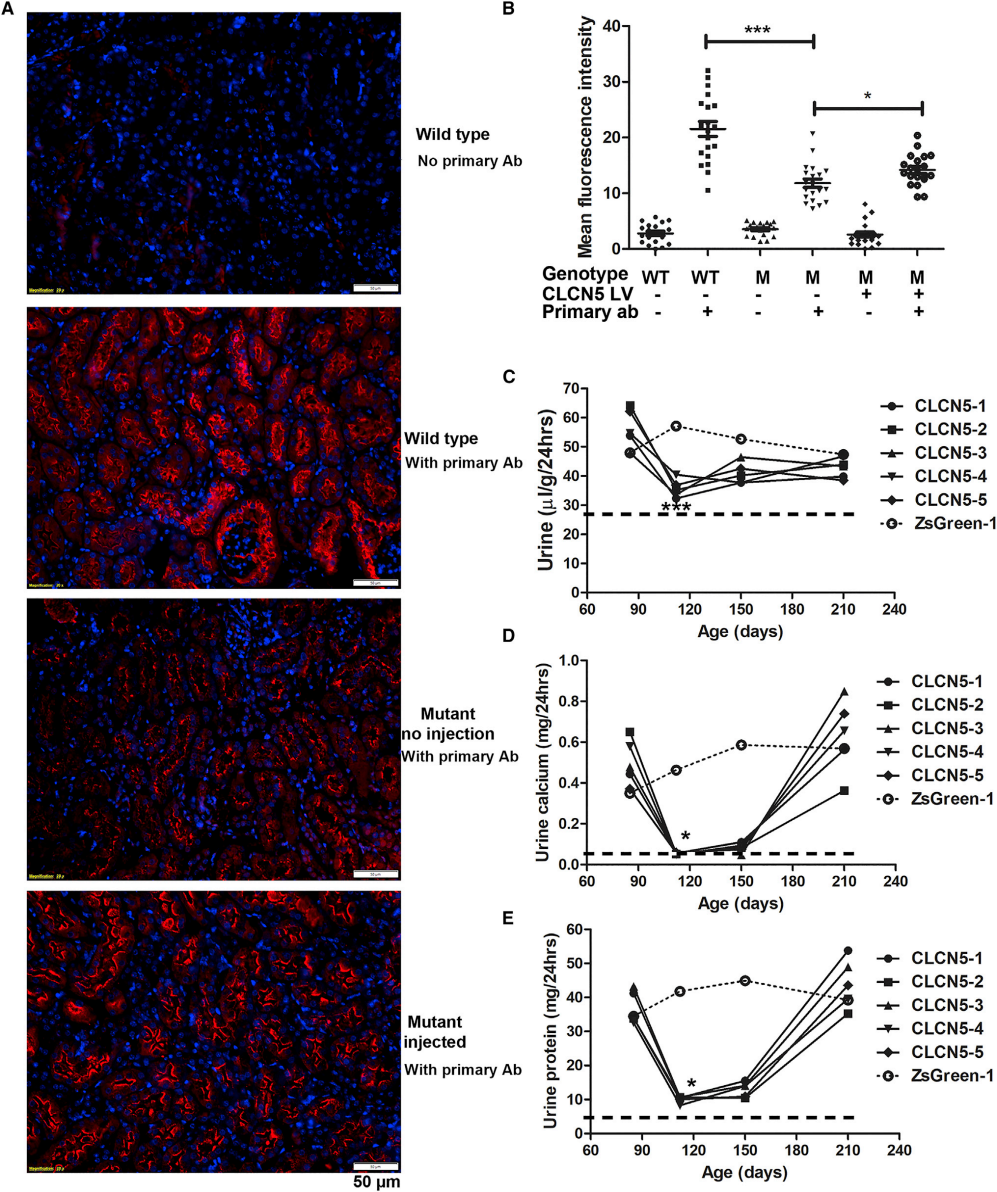
Therapeutic effects of *CLCN5* LV gene therapy (A) Immunofluorescence analysis of megalin expression in mutant mice with and without *CLCN5* LV delivery. Nuclei were stained with DAPI (shown in blue). Megalin signals are shown in red. (B) Quantitative analysis of tubular mean fluorescence intensity by ImageJ software. Each dot represents data from one tubule. Tubules from two kidneys (two sections per kidney) were analyzed. ∗p < 0.05 and ∗∗∗p < 0.0001 between the indicated two groups (Mann-Whitney test). (C) Effects of *CLCN5* LV delivery on diuresis in mutant mice. (D) Effects of *CLCN5* LV delivery on urinary calcium in mutant mice. (E) Effects of *CLCN5* LV delivery on urinary protein in mutant mice. For (C)–(E), all mutant mice received 280 ng p24 of *CLCN5* or ZsGreen LV to the left kidney at the age of 87 days. Data from each mouse are presented. The first data point is the time of LV injection and the pre-treatment urine parameters from urine samples were collected 37 days before LV injection. Post-treatment results are data from urine samples collected at the indicated ages. A dashed line indicates average values of wild-type male mice presented in Figure 1E. ∗p < 0.05 and ∗∗∗p < 0.001 compared with pre-treatment values (Dunn's multiple comparison test following Kruskal-Wallis test).

Considering that DD1 mainly affects male subjects, all subsequent gene therapy experiments described below were performed on male mice. We first examined whether restoring expression of ClC-5 in mutant mice can increase the expression of megalin, a protein involved in endocytosis that is deficient when ClC-5 deficiency is present.^35^, ^36^, ^37^ Consistent with reported observations, megalin expression was decreased in the kidneys of our mutant mice (Figure 5A). Two weeks after delivering 280 ng p24 of *CLCN5* LV into the kidneys of mutant mice, megalin expression was significantly increased, although still lower than in wild-type mice (Figures 5A and 5B).

**Figure 6:**
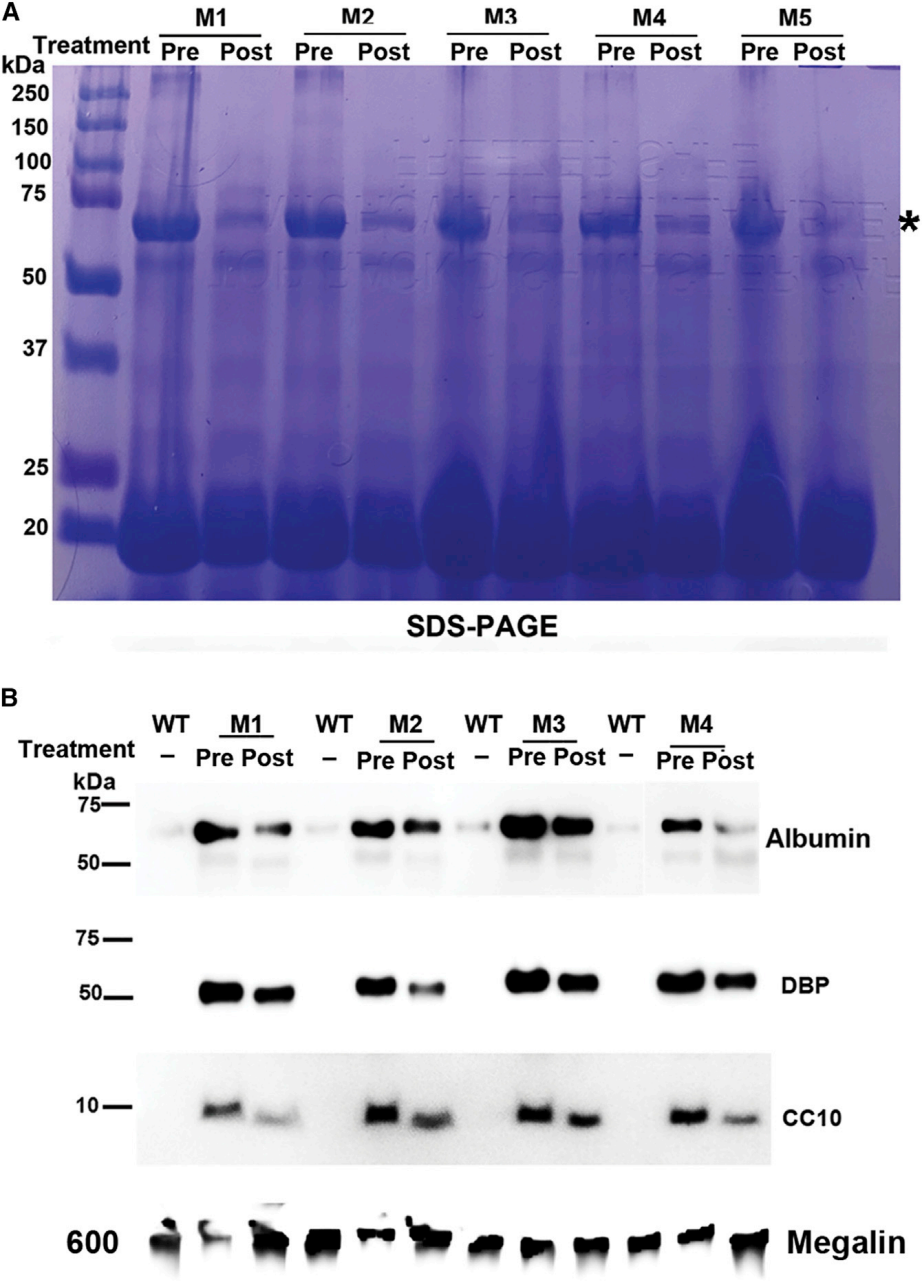
Analyses of urine protein after gene therapy (A) SDS-PAGE analysis of urinary proteins of mutant mice following *CLCN5* LV injection into the left kidney. ∗ indicates albumin. (B) Western blotting analyses of urinary marker proteins before and after *CLCN5* LV injection into the left kidney of mutant mice. For (A) and (B), pre- and post-treatment urine samples were collected 37 days before and 30 days after viral vector injection, respectively. Equal urine volumes were analyzed for each sample. M, mutant; WT, wild-type; DBP, vitamin D binding protein; CC10, club cell secretory protein.

We then tested whether delivering *CLCN5* LV to the kidneys of mutant mice could improve phenotypes. We first delivered 280 ng p24 of *CLCN5* LV to the left kidney of five mutant mice at the age of 87 days. A mutant mouse of similar age received ZsGreen LV to serve as a negative control. At 1 and 2 months after treatment, diuresis (Figure 5C), calciuria (Figure 5D), and proteinuria (Figure 5E) were all significantly improved. Daily urine volume and urinary protein excretion were still higher than those of wild-type mice (a dashed line indicates the averages of 2-month-old wild-type male mice). However, urinary calcium levels were similar to those of wild-type mice. Consistent with reduction of total urine protein after gene delivery, lower urinary protein was also evident in intensities of ∼20 kDa proteins and albumin (Figure 6A). Throughout our study, reduction of albumin co-occurred with improved diuresis and proteinuria. Thus, the intensity of urine albumin in SDS-PAGE analysis is a convenient and reliable indicator for gene therapy effects. Western blotting further confirmed reduced urine albumin, DBP, and club cell secretory protein (CC16, also called CC10) after delivering *CLCN5* LV to the left kidney (Figure 6B). The therapeutic effects largely disappeared at 4 months post-treatment (Figures 5C–5E). We also delivered *CLCN5* LV to the left kidneys of five mutant mice aged 62, 62, 66, 90, and 162 days, respectively. In every treated mouse, we observed a sharp decrease of urinary protein and urinary calcium excretion 1 month after *CLCN5* gene therapy (Figure S6). This decrease was not caused by aging of the mice, since aging was not associated with significant decreases in these parameters (Figure S7).

**Figure 7:**
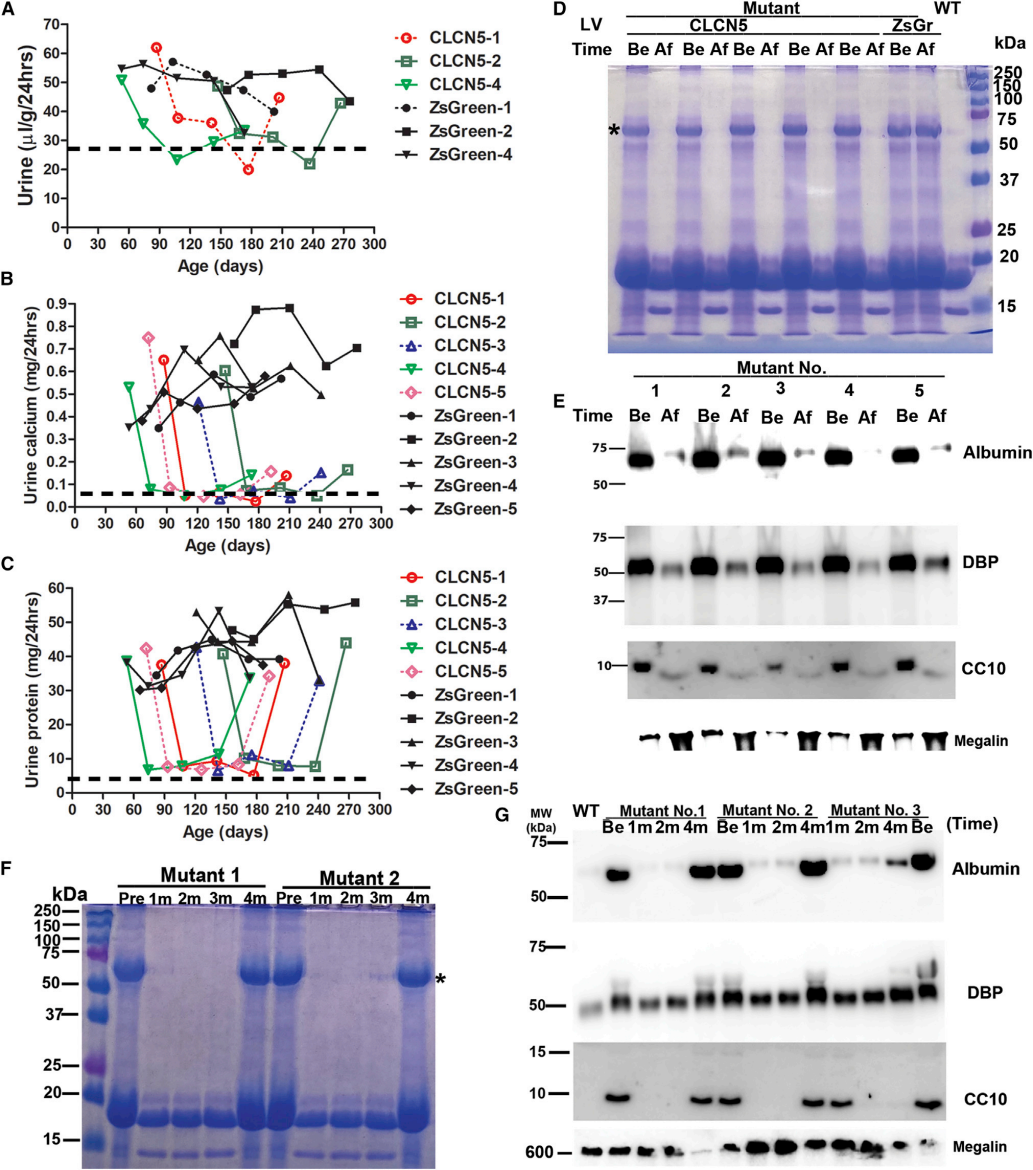
Therapeutic effects of delivering *CLCN5* LV into both kidneys (A) Effects of *CLCN5* LV delivery on diuresis in mutant mice. Age-matched mutant mice were injected with *CLCN5* LV or ZsGreen LV in both kidneys. Data from three of five pairs are presented here; data from the other two pairs are presented in Figure S8. (B) Effects of *CLCN5* LV delivery on urinary calcium of mutant mice. (C) Effects of *CLCN5* LV delivery on urinary protein of mutant mice. For (A)–(C), all mutant mice received 280 ng p24 of *CLCN5* or ZsGreen LV to both kidneys at ages of the first data points. Data from each mouse are presented. Pre-treatment urine samples were collected 27 days before LV injection. The age of the first data point for each mouse was the age of injection. Post-treatment urine samples were collected at the indicated ages. A dashed line indicates the values of wild-type male mice presented in Figure 1E. (D) SDS-PAGE analysis of urinary proteins of mutant mice following *CLCN5* LV injection into both kidneys. (E) Western blotting analyses of urinary marker proteins before and after *CLCN5* LV injection into both kidneys of mutant mice. For (D) and (E), post-treatment urine samples were collected 1 month after viral vector injection. Equal urine volumes were analyzed for each sample. (F) SDS-PAGE analysis of urine proteins pre- and post-treatment. (G) Western blotting analyses of urinary marker proteins pre- and post-treatment. Be, before treatment; Af, after treatment; 1m, 2m, and 4m indicate 1, 2, and 4 months after treatment, respectively.

We then performed another experiment, in which both kidneys of mutant mice were treated with *CLCN5* LV, each *CLCN5* LV-treated mouse had an age-matched mutant mouse treated with ZsGreen LV in both kidneys. The pairs had the ages of 53, 66, 82, 121, and 147 days, respectively. At 1, 2, and 3 months after treatment, every *CLCN5* LV-treated mouse showed greatly improved diuresis (Figure 7A and S8), calciuria (Figure 7B), and proteinuria (Figure 7C), with levels close to those of wild-type mice. On the contrary, ZsGreen LV-treated mice showed no improvement in these parameters. Again, reduced urinary protein 1 month after *CLCN5* LV treatment was confirmed by SDS-PAGE (Figure 7D) and western blotting (Figure 7E) analyses.

Four months after both kidneys were treated with *CLCN5* LV, diuresis and proteinuria returned to pre-treatment status (Figures 7A and 7C), whereas calciuria was still improved compared with pre-treatment levels and ZsGreen LV-treated mice (Figure 7B). In addition, we treated both kidneys of five mutant mice (81, 94, 110, 149, and 196 days old, respectively) with *CLCN5* LV, again all of these mice responded to the therapy (Figure S9). In these mice, urine calciuria was still improved 4 months after treatment, but returned to pre-treatment levels 6 months after treatment (Figure S9B). Timing of *CLCN5* gene therapy seemed to have little influence on therapeutic effects. Consistent with biochemical assays, SDS-PAGE (Figure 7F) and western blotting (Figure 7G), analyses of urine proteins also revealed that urine protein levels were reduced 1 and 2 months after treatment, but returned to pre-treatment levels 4 months after treatment.

### Immune rejection most likely caused the loss of therapeutic effects

**Figure 8:**
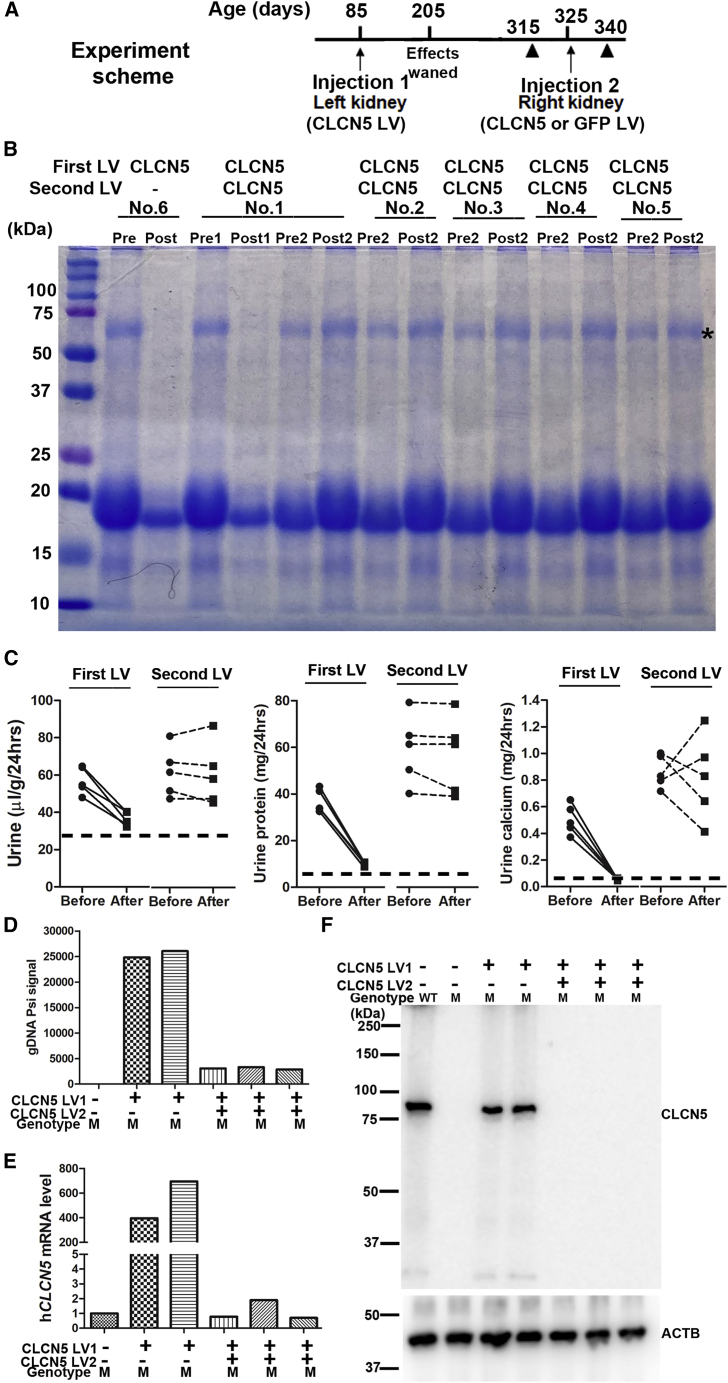
Delivery of a second dose of LV suggests involvement of immune responses to LV-mediated ClC-5 protein (A) Scheme of the experiment. Solid triangles indicate the times for urine collection before and after the second LV injection. (B) SDS-PAGE analysis of urinary proteins. All mice were male mutants. Mouse no. 6 was a naive mouse receiving the first dose of *CLCN5* LV; mice 1–5 were male mutant mice receiving a second *CLCN5* LV dose 8 months after the first dose. Pre1, before first *CLCN5* LV injection; Post1, after first *CLCN5* LV injection; Pre2, before second *CLCN5* LV injection; Post2, after second *CLCN5* LV injection. (C) Effects of first and second doses of *CLCN5* LV injection on diuresis (left), urinary protein (middle), and urinary calcium (right) excretion. Data were from the same five mice receiving the first and second *CLCN5* LV injection. The dashed horizontal lines indicate the values of wild-type mice. (D) Detecting vector DNA after first (*CLCN5* LV1) and second *CLCN5* LV (*CLCN5* LV2) injection using qPCR. (E) Detecting h*CLCN5* mRNA expression after first and second vector injections using qRT-PCR. (F) Detecting ClC-5 protein expression after first and second *CLCN5* LV injections by western blotting. The first four lanes of this image, serving as positive and negative controls, were from Figure 4A since these samples were analyzed on the same blot. M, mutant; WT, wild type.

There could be several possible causes for the loss of therapeutic effects 3 months after treatment: (1) promoter silencing; (2) epithelial cell aging and replacement; and (3) immune responses to the transgene product ClC-5 protein. We performed experiments to find the most likely causes. We injected *CLCN5* LV or GFP LV to the untreated right kidney of mutant mice 8 months after receiving a first injection of *CLCN5* LV in the left kidney (Figure 8A), after the therapeutic effects of the first dose were lost. Fifteen days after the second *CLCN5* LV injection, urine protein was not reduced in any of the five treated mice (animal nos. 1–5), although it was obviously reduced in these mice after the first dose of *CLCN5* LV (Figure 8B). Diuresis, proteinuria, and hypercalciuria all improved after the first injection but not the second one (Figure 8C). A naive mutant mouse receiving parallel treatment with the pre-injected mice showed improvement in all parameters assayed (Figure 8B; sample no. 6). Consistent with the lack of therapeutic effects following a second injection of *CLCN5* LV in pre-treated mice, LV DNA (Psi signal) (Figure 8D; see Table S11 for MIQE report), human *CLCN5* mRNA (Figure 8E; see Table S12 for MIQE report), and ClC-5 protein (Figure 8F) were barely detected or undetected in the kidneys of mice receiving the second *CLCN5* LV injection, but were readily detected in kidneys of the naive mouse 2 weeks after *CLCN5* LV injection. While mice pre-treated with *CLCN5* LV had no ClC-5 expression following a second *CLCN5* LV injection, delivering GFP LV to mice pre-treated with *CLCN5* LV resulted in robust GFP expression (Figure S10; *CLCN5*-LV, GFP-LV, nos. 2–4). GFP LV DNA was also detectable in the kidneys of GFP LV-injected mice without (Figure 3E; mouse GFP-LV, no. 1) and with previous *CLCN5* LV injection (Figure 3E; mice *CLCN5*-LV, GFP-LV, nos. 2–4). Expression of GFP but not ClC-5 after LV injection in mice pre-treated with *CLCN5* LV suggests that immune response to LV-expressed ClC-5, rather than the vector proteins, was most likely the reason for loss of therapeutic effects with time.

## Discussion

We used CRISPR-Cas9-mediated gene mutagenesis to generate a *Clcn5* mutant mouse model with 95% of the coding region deleted. We observed more severe phenotypes than observed in published models with p.Glu211Ala substitution^7^ or with exons 5 and 6 replaced.^26^^,^^27^ One unexpected phenotype was the partial embryonic or perinatal lethality of mutant mice in a C57/BL6 background. Since this phenotype was observed in pups of all 3 female founders, and no unintended mutations were detected in all 13 predicted off-targets analyzed, especially in the 4 potential off-targets on X chromosome, we reasoned that lethality was most likely caused by complete knockout of the *Clcn5* gene. This observation suggests that the *Clcn5* gene may function during early development, consistent with observations of *Clcn5* expression during embryonic development and in organs other than the kidney.^38^

Our mutant mice showed more severe proteinuria and hypercalciuria compared with published models.^7^^,^^26^^,^^27^ There are no other predicted genes (including non-coding genes) within 40 kbp surrounding the deleted region. Thus, the observed phenotypes could result from deleting 95% of the *Clcn5* coding region, which eliminated the possibility of expressing a partially functional ClC-5 protein. Our null mutant mice may be useful models to study the physiologic consequences of complete lack of ClC-5 protein.

Our data show that gene supplementary therapy could be an effective treatment option for DD1. We administered *CLCN5* LV vectors to 21 mutant mice (10 mice were treated in 1 kidney and 11 mice were treated in both kidneys). All treated mice showed significant improvement in diuresis, proteinuria, and hypercalciuria. After treatment, diuresis and urinary calcium levels were restored to near normal values, whereas urinary protein levels were reduced to 20% of pre-treatment levels, although they were still 80% higher than normal levels. Thus, gene therapy was effective in ameliorating the symptoms of DD1. Furthermore, mice aged 53–196 days responded similarly to the therapy, indicating that the timing of gene therapy was not critical. This observation could be relevant for clinical situations, since not only young patients might benefit from gene therapy.

*Clcn5* is also expressed in the intestinal epithelium.^39^ One study raised the possible role of intestinal calcium absorption in hypercalciuria of ClC-5-deficient mice.^31^ We delivered the *CLCN5* LV into the kidney by retrograde ureter injection and restored urinary calcium levels in mutant mice. Our data suggest that ClC-5 expressed in the kidney plays a major role in calcium maintenance.

Over 50% of DD1 cases are caused by changes predicted to express no full-length ClC-5 protein. Effectively ameliorating DD1 phenotypes in ClC-5 null mice by gene supplementary therapy suggests that such therapy most likely will benefit these patients. About 33% of DD1-causing variants are missense ones that express unstable, dislocated, or dysfunctional ClC-5 proteins.^40^^,^^41^ Gene therapy may benefit some of those subjects expressing unstable or dislocated ClC-5 proteins. It remains to be determined to what extent gene therapy will benefit those subjects expressing a malfunctioned ClC-5, since ClC-5 most likely forms a homodimer,^28^^,^^29^ and the endogenous malfunctioned ClC-5 protein might interfere with the function of the exogenous ClC-5 protein.

Immune responses to transgene products have been reported in animal studies,^42^^,^^43^ as well as in gene therapy clinical trials for α-1-antitrypsin deficiency^44^ and Duchenne’s muscular dystrophy.^45^ In this study, effects of *CLCN5* gene therapy effects lasted for up to 3 months but diminished after 4 months. Our experiments suggested that immune responses to the transgene product, ClC-5 protein, most likely caused the loss of gene therapy effects. This reasoning is supported by our observations that GFP LV, but not *CLCN5* LV, could be successfully delivered into kidneys of mice previously treated with *CLCN5* LV and mediate transgene expression. On the other hand, if the loss of therapeutic effects had been caused by factors unrelated to immune responses, such as promoter silencing and cell replacement, we would expect ClC-5 expression and improvement of phenotypes following the second *CLCN5* LV injection, as we have observed after the first *CLCN5* LV injection. Thus although more work is needed to demonstrate immune responses to ClC-5 protein, avoiding immune responses to ClC-5 protein could be one of the strategies to prolong therapeutic effects.

In this study, we used human *CLCN5* rather than mouse *Clcn5* cDNA to restore ClC-5 expression in our mouse models. To our ClC-5 null mice, human and mouse cDNA make no difference since they do not express ClC-5 protein. In the 50% of DD1 patients who express truncated or no ClC-5 protein, immune responses to the transgene product ClC-5 protein could pose a challenge in developing DD1 gene therapy. Their lack of full-length ClC-5 protein makes it difficult to enhance ClC-5 function by small-molecule drugs, so gene supplementary therapy could be the best option. Minimizing immune responses to the transgene product in such patients could help to achieve sustained therapeutic effects.

One strategy to minimize immune responses is to use tissue-specific promoters to avoid expression of ClC-5 in dendritic cells (DCs), which mediate adaptive immune responses.^42^^,^^46^ DCs in the renal tubulointerstitium^47^ can be transduced by the LV vectors to elicit adaptive immune responses to the transgene. In this study, we used the EF1 alpha promoter, active in essentially all cells, for proof of concept. Since proximal tubules are the main location of reabsorbing,^5^, ^6^, ^7^, ^8^, ^9^ using tubule proximal cell-specific promoters, such as those for *Npt2a*^48^ or *Sgtl2*,^49^ may help to reduce immune responses. Another strategy is to further reduce expression of the transgene in DCs by incorporating the miR-142-3p target sequence in the 3ʹ untranslated region of the transgene.^43^^,^^50^ DCs express high levels of miR-142-3p, which will inhibit transgene expression in these cells. This strategy has been successfully used to minimize immune responses to factor VIII in hemophilia gene therapy.^51^ Co-delivering ImmTOR (a lipid nanoparticle with rapamycin) and an AAV vector inhibited immune responses to the vector and allowed repeated delivery.^52^ Thus, co-delivery of ImmTOR and *CLCN5* LV vectors might inhibit transgene-induced immune response and elongate the duration of therapeutic effects. Finally, delivering *CLCN5* LV to neonatal mice may avoid immune responses and achieve long-term therapeutic effects. Although immune response to ClC-5 might have caused the loss of therapeutic effects, other mechanisms, such as promoter silencing and cell replacement, also cannot be excluded.

Changes in various other genes cause renal tubular disorders, affecting normal functions of the proximal tubule (DD1 and DD2), the loop of Henle (Bartter syndrome, familial hypomagnesaemia with hypercalciuria), the distal convoluted tubule (Gitelman syndrome, Gordon syndrome), and the collecting duct (Liddle syndrome, apparent mineralocorticoid excess syndrome, pseudohypoaldosteronism type 1, and hereditary distal renal tubular acidosis).^53^ Developing gene therapy for DD1 could benefit development of treatments for other tubular disorders, for which there are no treatments targeting the etiology and stopping disease progression. To the best of our knowledge, our work is the first to show that the retrograde ureter delivery method can efficiently deliver viral vectors into tubule cells and greatly improve tubular function in a disease model.

## Materials and methods

### Study approval

Experiments were conducted in accordance with the National Research Council Publication Guide for Care and Use of Laboratory Animals, and approved by the Institutional Animal Care and Use Committee of Wake Forest University Health Sciences (Animal protocol number A19-053). Mice were kept in micro-isolator cages with 12-h light/dark cycles and were fed ad libitum. Carbon dioxide (CO_2_) overdose, which causes rapid unconsciousness followed by death, was used to euthanize mice. Mice were exposed to CO_2_ without being removed from their home cage, so that they were not stressed by handling or being moved to a new environment. The CO₂ flow rate was set to displace 10%–30% of the cage volume per minute. When mice showed deep narcosis, they were subjected to cervical dislocation as a secondary method of euthanasia. After euthanasia, kidney tissues were collected for further analyses.

### DNA constructs

LV plasmid pCSII-hCLCN5 was constructed to express codon-optimized human *CLCN5* cDNA under the control of human EF1 alpha promoter. Plasmid pCSII-hCLCN5 was made by replacing the XhoI-XbaI fragment of pCSII-EF-miRFP709-hCdt (1/100)^54^ (a gift from Vladislav Verkhusha, Addgene plasmid no. 80007; http://n2t.net/addgene:80007; RRID: Addgene_80007) with a synthesized and codon optimized cDNA encoding for human ClC-5 protein (see Table S13 for cDNA and protein sequences). Gene synthesis was performed by GenScript and the sequence was confirmed by Sanger sequencing. Plasmids pMD2.G (Addgene plasmid no. 12259; http://n2t.net/addgene:12259; RRID: Addgene_12259), pMDLg/pRRE (Addgene plasmid no. 12251; http://n2t.net/addgene:12251; RRID:Addgene_12251) and pRSV-Rev (Addgene plasmid no. 12253; http://n2t.net/addgene:12253; RRID:Addgene_12253) were gifts from Didier Trono and have been described previously.^55^ The ZsGreen- and GFP-expressing lentiviral transfer plasmids pLVX-IRES-ZsGreen1 and CmiR0001-MR03 were purchased from Takara Bio and GeneCopoeia, respectively. Sequence information for primers is listed in Table S14.

### Generation of ClC-5 null mice

ClC-5 null mutant mice were generated by CRISPR-Cas9-mediated knockout of the mouse *Clcn5* gene. Three sgRNAs, targeting mouse *Clcn5* intron 2 (gRNA1: UCUGGGUUGAUCAUCUAAAC), intron 4 (gRNA2: AGGGGGCCGAAUUCUUGCAA), and exon 12 (gRNA3: GCAAUGCUAACUAGUAGACG), respectively, were analyzed by CRISPRater,^56^ and injected into fertilized mouse eggs with *Streptococcus pyogenes* Cas9 (SpCas9) mRNA to generate targeted knockout offspring. Three sgRNAs were used to increase the efficiency of deleting a large DNA region. RNA microinjection into fertilized eggs was done at Cyagen (Biotechnology Company, Santa Clara, CA). F0 founder animals were identified by PCR followed by sequence analysis, and then mated with wild-type female mice to generate F1 animals. The founder heterozygous F1 mice in a C57/BL6 background were subsequently housed in the pathogen-free animal facility at Wake Forest University Health Sciences. To avoid partial embryonic or perinatal lethality of mutant mice in the C57/BL6 background, C57/BL6 heterozygous female mice were mated with FVB/NJ wild-type males to obtain mutant male mice. Carrier male and female mice in 50% C57/BL6 and 50% FVB backgrounds were mated to generate mutant female mice.

### Genotyping of mutant mice

Tail or ear snips were digested with proteinase K (400 μg/mL) in Taq DNA polymerase buffer (New England Biolabs) containing 0.45% NP40 at 55°C for 3 h to overnight. Proteinase K was inactivated at 95°C for 10 min. The cleared lysate was directly used for polymerase chain reaction (PCR) amplification. PCR primers CLCN5-KF2 and CLCN5-KR2 were used to amplify a product of about 1,000 bp from the mutant allele. CLCN5-KF2 and CLCN5-W2 were used to amplify a product of 540 bp from the wild-type allele. PCR cycling included an initial denaturation at 94°C for 5 min, followed by 35 cycles of denaturation at 94°C for 30 s, annealing at 60°C for 30 s, and extension at 72°C for 60 s/kb, and a final extension step at 72°C for 5 min. PCR will amplify the 540 bp product in wild-type mice, both the 540 bp and the 1,000 bp products in heterozygous mice, and the 1,000 bp product in homozygous mutant mice with the two pairs of primers.

### Off-target analyses

Potential off-targets for each of the sgRNAs were predicted by the target prediction tool CCTop^57^ and CRISPOR.^30^ Predicted off-target sites with up to one mismatch in the seed and three or four total mismatches are listed in Tables S3–S5. The coordinates of off-targets were based on the GRCm38/mm10 assembly (2011). Genomic DNA of male mutant mice was amplified by PCR primers designed for the following off-targets: the only off-target hitting an exon, the four off-targets on the X chromosome, all five off-targets with 3-nt mismatches to the sgRNAs, and two off-targets with 4-nt mismatches to the sgRNA and high predicted scores. The amplified DNA samples were subjected to Sanger sequencing and the sequences were compared with the mouse reference sequences.

### Isolation and culture of kidney proximal tubule cells

Kidney proximal tubule cells were isolated as reported previously.^58^ Kidney cortices were minced and incubated with collagenase (Worthington Biochemical, Freehold, NJ) and soybean trypsin inhibitor (GIBCO Laboratories, Grand Island, NY), both at concentrations of 0.5 mg/mL for 30 min. After large undigested fragments were removed by gravity, the suspension was mixed with an equal volume of 10% horse serum in Hank’s solution and then centrifuged at 500 revolutions/min for 7 min at room temperature. The pellets were washed once by centrifugation and then suspended in serum-free cell culture medium, which was a mixture of Dulbecco’s modified Eagle’s medium and Ham’s F-12 nutrient mixture (1:1) containing 2 mM glutamine, 15 mM N-2-hydroxyethylpiperazine-N-2-ethanesulfonic acid, 500 U/mL penicillin, and 50 pg/mL streptomycin. The pelleted tissue pieces were resuspended in high-glucose DMEM medium containing 10% FBS, 1% L-glutamine (cat. no. SH30034, HyClone Laboratories, South Logan, UT), and 1% penicillin-streptomycin supplement (HyClone Laboratories, cat. no. SV30010), and incubated in tissue culture dishes at 37°C 5% CO_2_ for epithelial cells to grow out of the tissues and attach to the dish bottom. After two passages, the cells were dissociated by trypsinization and seeded into 24-well plates at 8 × 10^4^ cells/well for LV transduction.

### LV production

Lentiviral transfer plasmid pCSII-hCLCN5, CmiR0001-MR03, and pLVX-IRES-ZsGreen1 were used to produce LVs expressing ClC-5, GFP, and ZsGreen, respectively, with a third-generation packaging system. In brief, 13 million actively proliferating HEK293T cells in 15-cm dishes were changed into 15 mL Opti-MEM. The following were used for co-transfection: 12 μg lentiviral transfer plasmid DNA (pCSII-hCLCN5, CmiR0001-MR03, or pLVX-IRES-ZsGreen1), 14 μg pMDLg/pRRE, 6 μg pMD2.G, and 4 μg pRSV-Rev. The DNA was mixed in 1 mL Opti-MEM. In a separate tube, 108 μL polyethylenimine (1 mg/mL, PEI, Synchembio, cat. no. SH-35421) was added in 1 mL Opti-MEM. The DNA and PEI mixtures were then combined and incubated at room temperature for 15 min. The DNA/PEI mixture was then added to the cells in Opti-MEM. Twenty-four hours after transfection, the medium was changed to 15 mL Opti-MEM and the LVs were collected 48 and 72 h after transfection. The combined supernatants were spun for 10 min at 500 × *g* to remove cellular debris. The cleared supernatant was further processed as described below for *in vivo* delivery.

### Concentrating LVs

The supernatant containing LVs was first concentrated with the KR2i TFF System (KrosFlo Research 2i Tangential Flow Filtration System, Spectrum Lab, cat. no. SYR2-U20) using the concentration-diafiltration-concentration mode. In brief, 150–300 mL supernatant was first concentrated to about 50 mL, diafiltrated with 1,000 mL PBS, and finally concentrated to about 8 mL. The hollow fiber filter modules were made from modified polyethersulfone, with a molecular weight cutoff of 500 kDa. The flow rates and pressure limits were 80 mL/min and 8 psi for the filter module D02-E500-05-N, and 10 mL/min and 5 psi for the filter module C02-E500-05-N, respectively.

To further increase the vector concentration for *in vivo* delivery, four volumes of TFF concentrated vectors were laid on one volume of 10% sucrose buffer (in 50 mM Tris-HCl [pH 7.4], 100 mM NaCl, 0.5 mM EDTA). The viral vectors were centrifuged at 10,000 × *g* at 4°C for 4 h and re-suspended in ∼0.5 mL PBS. The vectors were aliquoted into 100 μL/tube and frozen at −80°C for future use.

### LV quantification

Concentrations of LVs were determined by p24 (a capsid antigen)-based ELISA (Cell Biolabs, QuickTiter Lentivirus Titer Kit, cat. no. VPK-107). Concentrated vectors were diluted 200-fold for assays. To assay non-concentrated samples, viral particles were precipitated according to the manufacturer’s instructions such that the soluble p24 protein was not detected.

### LV transduction *in vitro*

Non-concentrated and concentrated LVs (equivalent to 10–50 ng p24 protein) were added to 2.5 × 10^4^ cells grown in 24-well plates, with 8 μg/mL polybrene. The cells were incubated with the vector-containing medium for 12–24 h, after which the medium was replaced with normal medium. Seventy-two hours after transduction, the cells were collected for gene expression analyses.

### Retrograde ureteral injection

LVs were delivered to the kidney by retrograde ureteral injection as previously reported.^18^, ^19^, ^20^, ^21^, ^22^, ^23^ Mice were anesthetized with 3% isoflurane inhalation and the left kidney was exposed via a 2-cm flank incision and gently separated from the surrounding fat. An atraumatic vascular clip (S&T Vascular Clamps, cat. no. 00400-03, Fine Science Tools, Heidelberg, Germany) was placed on the ureter below the injection site to prevent leakage to the bladder. Using a 30-gauge 0.5-inch needle connected to a 1-mL syringe, lentiviral particles were injected into the ureter just below the ureteropelvic junction. The total volume of viral solution did not exceed 100 μL. The concentration of the viral vectors was 2–4 ng/μL. After 15 min, the clamp was removed and the surgical site was closed in two layers (first muscle, then skin) with an absorbable 5-0 Vicryl suture. If bilateral injections were performed, the same procedure was repeated on the right kidney after the closure of the left incision. Immediately after surgery and before mice awakened, they received 5–10 mg/kg carprofen. Together with the first carprofen dose, they also received buprenorphine SR (0.5–1.0 mg/kg), both via subcutaneous injection. The mice received 5–10 mg/kg carprofen again 24 and 48 h after the surgery. The mice were singly housed after waking up from the surgery to prevent wound damage by cage mates.

### Urine collection

Mice were housed in Hatteras Instruments Model MMC100 metabolic mouse cages (Hatteras Instruments, Cary, NC) for 24 h for urine collection. The urine samples were briefly spun at 1,000 × *g* for 5 min to remove possible particles. Urine volume was measured using a 200-μL pipette.

### Urine biochemistry

Urinary calcium concentrations were determined using Calcium Assay Kits (Colorimetric) (ab102505, AbCam). Urine samples from wild-type and *CLCN5* LV-treated mice were diluted 3.6-fold, and those from untreated mutant mice were diluted 10-fold with water before assay. Total calcium excretion was calculated by multiplying the calcium concentration by the respective urine volume collected during 24 h. Urinary total protein concentration was determined using Pierce BCA Protein Assay Kits (cat. no. 23225). All urine samples were diluted 10-fold with water before protein assays. Total urinary protein excretion was calculated by multiplying the urine protein concentration by the respective urine volume collected during 24 h. Urinary creatinine was diluted 10-fold with saline and then assayed using Mouse Creatinine Assay Kits (Crystal Chem, no. 80350). All measurements were performed according to the manufacturers’ instructions.

### SDS-PAGE and western blotting analyses

Kidney tissues were lysed in RIPA buffer with protease inhibitors (0.5 mm PMSF and 1x cOmplete Protease Inhibitor Cocktail, Roche Diagnostics, Indianapolis, IN) and phosphatase inhibitors (50 mM NaF, 1.5 mM Na3VO_3_), and the lysates were mixed with Laemmli buffer for SDS-PAGE for western blotting analyses. Cultured cells and urine samples were lysed directly in 1x Laemmli buffer containing protease inhibitors and phosphatase inhibitors. Anti-β-actin antibody was from Sigma (A5441, 1:5,000; St. Louis, MO), ClC-5 Rabbit polyclonal antibody from GeneTex (GTX53963, 1:500, Irvine, CA), CC16 rabbit polyclonal antibody from BioVendor (RD181022220-01, 1:500, Asheville, NC), albumin goat polyclonal antibody from Bethyl Laboratories (A80-129A, 1:1,000, Montgomery, TX), DBP rabbit polyclonal antibody from Proteintech (16922-1-AP, 1:1,000, Rosemont, IL), megalin rabbit polyclonal antibody from Abcam (ab76969, Boston, MA), and GFP antibody was from Cell Signaling Technology (Danvers, MA, cat. no. 2555S). HRP-conjugated anti-mouse IgG (H + L) (Thermo Fisher Scientific, cat. no. 31430, 1:5,000) and anti-rabbit IgG (H + L) (cat. no. 31460, 1:5,000) secondary antibodies were used in western blotting. Chemiluminescence reagents (Thermo Fisher Scientific) were used to visualize the protein signals under an LAS-3000 system (Fujifilm, Tokyo, Japan).

### Protein mass spectrometry analysis of urinary proteins

To identify protein from urine of mutant mice, urine from wild-type and mutant male mice were separated by SDS-PAGE and stained by Coomassie blue. The intense bands around 61 kDa observed in mutant urine but not in wild-type urine samples were sliced out and sent for protein mass spectrometry analysis (MSBioworks, Ann Arbor, MI). Samples digested by trypsin were analyzed by Nano LC-MS/MS with a Waters M-Class HPLC system interfaced to a Thermo Fisher Fusion Lumos mass spectrometer. Peptides were loaded on a trapping column and eluted over a 75-μm analytic column at 350 nL/min; both columns were packed with Luna C18 resin (Phenomenex). The mass spectrometer was operated in data-dependent mode, with the Orbitrap operating at 60,000 FWHM and 15,000 FWHM for MS and MS/MS, respectively. The instrument was run with a 3-s cycle for MS and MS/MS. Data were searched using a local copy of Mascot (Matrix Science).

### Immunofluorescence analyses

Kidney tissues were fixed in 4% paraformaldehyde/PBS at 4°C overnight. Some tissues were embedded in OCT for cryosections, and some were dehydrated and embedded in paraffin. Paraffin-mounted sections of 5–8 μm were prepared for histologic and immunofluorescence analyses. For immunofluorescence staining, deparaffinized and rehydrated sections were incubated with primary antibodies (1:200 for ClC-5 and megalin antibodies, 1:1,000 for GFP antibody) following blocking, and were then incubated with CF-594-conjugated secondary antibody (Biotium, Fremont, CA). Sections were mounted in mounting medium with DAPI (Vector Laboratories, Burlingame, CA). Images were acquired with an Axio M1 microscope equipped with an AxioCam MRc digital camera (Carl Zeiss, Thornwood, NY). Different images were assembled into one file with Adobe Photoshop, with subsequent resizing, rotation, and cropping. Fluorescence intensity was analyzed by NIH ImageJ software (v.1.49).

### Vector DNA detection

Each kidney was first cut into two longitudinal parts along the middle plate, and then each part was divided into six pieces of similar mass by slicing in the direction perpendicular to the original cut. Pieces were stored at −80°C for DNA, RNA, and protein extraction. One of the pieces was used for genomic DNA isolation using a DNeasy Blood & Tissue Kit (QIAGEN, Germantown, MD). To detect LV DNA, the Psi or GFP sequence from the LV was detected by qPCR, using respective primers and SYBR Green Master Mix (Thermo Fisher Scientific). Mouse *Gapdh* was used as the reference gene, with TaqMan Universal PCR Master Mix and *Gapdh* hydrolysis probe (Thermo Fisher Scientific) used for qPCR detection. Minimum Information for Publication of Quantitative Real-Time PCR Experiments (MIQE) guidelines were followed.^59^

### RNA isolation and qRT-PCR analyses

A miRNeasy Mini Kit (cat. no. 217004, QIAGEN) was used to isolate total RNA from tissues and cultured cells. The QuantiTect Reverse Transcription Kit (QIAGEN) was used to reverse-transcribe the RNA to cDNA. qRT-PCR was run on a QuantStudio3 or ABI 7500 instrument with primers listed in Table S14.

### Statistical analysis

Statistical assessments were performed on urinary parameters and immunostaining data using GraphPad Prism (v.5) software. Data are presented as mean ± standard error of the mean (SEM). For analyses comparing two groups, Mann-Whitney tests were performed. When more than two groups were compared, Dunn's multiple comparison tests were performed following Kruskal-Wallis tests. Significance was set at ∗p *<* 0.05, ∗∗p *<* 0.01, and ∗∗∗p *<* 0.001.

### Data availability

Plasmids and sequence information are available upon request.

## Supplemental information

Document S1. Figures S1–S10 and Tables S1–S14

Document S2. Article plus supplemental information
