# Supplementary material for: Lentiviral vector mediated gene therapy for type I Dent disease ameliorates Dent disease-like phenotypes for three months in ClC-5 null mice
Source: Mol Ther Methods Clin Dev. 2022 Sep 24;27:149–66. doi: 10.1016/j.omtm.2022.09.009 (PMC9574595; doi:10.1016/j.omtm.2022.09.009)
Supplement: Document S1. Figures S1–S10 and Tables S1–S14 [file mmc1.pdf]

OMTM, Volume *27*

Supplemental information

Lentiviral vector mediated gene therapy for type I Dent disease ameliorates Dent disease-like phenotypes for three months in ClC-5 null mice

Manish Kumar Yadav, Kyung Whan Yoo, Anthony Atala, and Baisong Lu

Table S1. Predicted off-targets for sgRNA 1

Coordinates	strand	MM	target_seq	PAM	distance	Intron or exon	gene name	gene id	Off-target name
chrX:7185297-7185319	-	0	TCTGGGTTGATCATCTAAAC	TGG	865	I	Clcn5	ENSMUSG00000004317	
chr6:54711115-54711137	+	3	TTTAGGTAGATCATCTAAAC	TGG	7264	-	Mturn	ENSMUSG00000038065	Off-1-1
chr17:85954799-85954821	+	4	ACAGGGCTGAACATCTAAAC	TGG	29844	-	Srbd1	ENSMUSG00000024135	
chr17:85350804-85350826	+	3	TCTGGGGTTGTCATCTAAAC	AGG	40747	I	Camkmt	ENSMUSG00000071037	Off-1-2
chr5:65536652-65536674	+	4	TCAGCATTGATTATCTAAAC	AGG	115	I	Smim14	ENSMUSG00000037822	
chr1:105511230-105511252	-	4	TCTTGGTGATTCATCTAAAC	TGG	7170	I	Pign	ENSMUSG00000056536	
chr1:5468047-5468069	-	4	TCTAGTTGGATCACCTAAAC	TGG	62469	-	Gm38264	ENSMUSG00000102907	
chr16:3714939-3714961	+	4	GCTGTGTTGTTCACCTAAAC	TGG	232	I	Mefv	ENSMUSG00000022534	
chrX:39778533-39778555	-	4	ACTGGGTTTCTCTTCTAAAC	AGG	18029	-	Gm14571	ENSMUSG00000083845	Off-1-4
chr3:43053460-43053482	-	3	TTTGGGTTGATGATATAAAC	AGG	NA	-	NA	NA	Off-1-3
chr3:154172243-154172265	+	4	TGTGGGTGGAACAGCTAAAC	AGG	626	I	Slc44a5	ENSMUSG00000028360	
chr7:99620983-99621005	-	4	CCTGAGTTGACCATTTAAAC	TGG	3077	-	Gm4980	ENSMUSG00000096606	
chr6:82418407-82418429	+	4	TCTGGAGTCATCATTTAAAC	TGG	13043	I	Tacr1	ENSMUSG00000030043	
chr13:82483478-82483500	-	4	ACTGGCTTTATCATCAAAAC	AGG	NA	-	NA	NA	
chrX:60831139-60831161	+	4	CCTGGGTGCATCATCCAAAC	AGG	12357	-	Gm14660	ENSMUSG00000081891	Off-1-5

The first row is the on-target information. Efficacy score by CRISPRater: 0.74 MEDIUM. The nucleotides in red indicate those mismatching with the sgRNA. The highlighted off-targets were sequenced in a male mutant. MM: number of mismatched nucleotides. The coordinates of off-targets were based on the GRCm38/mm10 assembly (2011).

Table S2. Predicted off-targets for sgRNA 2

Coordinates	strand	MM	target_seq	PAM	distance	Intron or exon	gene-name	gene-id	**Off-target name**
chrX:7171872-7171894	+	0	AGGGGGCCGAATTCTTGCAA	GGG	606	I	Clcn5	ENSMUSG00000004317	
chr6:118613497-118613519	-	4	AGGAACCCAAATTCTTGCAA	TGG	127	I	Cacna1c	ENSMUSG00000051331	off-2-3
chr18:39908859-39908881	+	4	CAGGGGCAGAAGTCTTGCAA	TGG	NA	-	NA	NA	
chr2:170951092-170951114	+	3	AGGAGGCCTAAATCTTGCAA	AGG	2965	-	Gm14263	ENSMUSG00000080955	off-2-1
chr16:75838076-75838098	-	4	AATGGGCCAAAATCTTGCAA	TGG	20695	-	Samsn1	ENSMUSG00000022876	
chr8:87839483-87839505	+	4	AGGTGTACGAATACTTGCAA	TGG	19779	I	Zfp423	ENSMUSG00000045333	
chr3:129811585-129811607	-	4	AGAGGTCGGAATCCTTGCAA	TGG	4	I	Rrh	ENSMUSG00000028012	
chr7:135990910-135990932	-	4	AAGGGTCCAAATGCTTGCAA	AGG	37127	-	Gm9341	ENSMUSG00000098031	
chr5:47135608-47135630	-	4	AGGGGCACCAAGTCTTGCAA	GGG	18021	-	Gm43601	ENSMUSG00000104660	
chr13:15618199-15618221	-	4	TGGGGCCTGAATTATTGCAA	TGG	4315	I	Gli3	ENSMUSG00000021318	
chr6:18834225-18834247	+	4	AGTGAGCCCAATTATTGCAA	TGG	9166	-	Gm5874	ENSMUSG00000071568	
chr5:144326148-144326170	-	4	AGAGGGCAGAGTCCTTGCAA	GGG	1516	I	Baiap2l1	ENSMUSG00000038859	
chr2:60634545-60634567	+	4	AGGGAGCCAACTGCTTGCAA	GGG	0	E	Itgb6	ENSMUSG00000026971	off-itgb6
chr16:34004735-34004757	-	4	TGGGGACCGCATTCCTGCAA	GGG	4513	I	Kalrn	ENSMUSG00000061751	
chr9:101701694-101701716	-	3	AGGGTGCCAAATTCTGGCAA	GGG	68656	-	Gm29521	ENSMUSG00000100807	off-2-2

The first row is the on-target information. Efficacy score by CRISPRater: 0.76 MEDIUM. The nucleotides in red indicate those mismatching with the sgRNA. The highlighted off-targets were sequenced in a male mutant. MM: number of mismatched nucleotides. The coordinates of off-targets were based on the GRCm38/mm10 assembly (2011).

Table S3. Predicted off-targets for sgRNA 3

Coordinates	strand	MM	target_seq	PAM	distance	Intron or exon	gene name	gene id	Off-target name
chrX:7158977-7158999	-	0	GCAATGCTAACTAGTAGACG	AGG	0	E	Clcn5	ENSMUSG00000004317	
chr16:65536691-65536713	+	4	TCAATATTACCTAGTAGACG	TGG	1686	-	Pou1f1	ENSMUSG00000004842	Off-3-1
chrX:106257785-106257807	-	4	ACAGTGTTAAGTAGTAGACG	GGG	2409	-	Fnd3c2	ENSMUSG00000073012	Off-3-2
chr9:62530943-62530965	+	4	TCAATCCCAACTACTAGACG	GGG	5688	I	Coro2b	ENSMUSG00000041729	Off-3-3
chr11:80937879-80937901	-	4	CCACTGCTAACAAATAGACG	TGG	14579	I	Asic2	ENSMUSG00000020704	
chrX:18446775-18446797	-	4	GGAATCCTGACTAGTATACG	TGG	781	I	4930578C19Rik	ENSMUSG00000037358	Off-3-4
chr2:75105387-75105409	-	4	CCAAGGCTAACTAATAGGCG	GGG	19462	-	Gm13652	ENSMUSG00000087333	
chr7:81835546-81835568	+	4	GCAAAGCTAAGTAGAAAACG	GGG	6115	-	Btbd1	ENSMUSG00000025103	
chr3:9472233-9472255	+	4	GCAAAGATGACTAGTAGACT	AGG	1115	I	Zfp704	ENSMUSG00000040209	
chr3:99318004-99318026	-	4	GGAATGTTAACCAGTAGACA	AGG	1646	I	Tbx15	ENSMUSG00000027868	
chr6:86859252-86859274	-	4	GCAATGACAGCTAGTAGACC	TGG	3599	I	RP24-467O4.3	ENSMUSG00000108216	
chr4:4847984-4848006	+	4	GCAATAATAACTAGAAGAAG	AGG	54629	-	Impad1	ENSMUSG00000066324	
chr18:28959114-28959136	+	4	GCAATTCTAGCCAGTAGACA	GGG	NA	-	NA	NA	
chr1:152795360-152795382	-	4	GCAAGGCTGACTGGTAGACA	GGG	4812	-	Ncf2	ENSMUSG00000026480	
chr3:109170448-109170470	+	4	GGAATGCTAACTCGTCGTCG	TGG	1991	-	Slc25a24	ENSMUSG00000040322	

The first row is the on-target information. Efficacy score by CRISPRater: 0.66 MEDIUM. The nucleotides in red indicate those mismatching with the sgRNA. The highlighted off-targets were sequenced in a male mutant. MM: number of mismatched nucleotides. The coordinates of off-targets were based on the GRCm38/mm10 assembly (2011).

Table S4. All off-targets for sgRNA1 predicted with CRISPOR.

Number of mismatch	Position	gene
3	intergenic	Mturn-Znrf2
4	intergenic	Six2-Srbd1
4	intergenic	Cetn3-Mir3961
4	intron	Smim14
4	intergenic	Gm715-Sox3
4	intron	LOC108167736
4	intron	Fig4
3	intron	Camkmt
4	intron	Rgs6
4	intergenic	Gm17634-Pign
4	intron	Gnaq
4	intergenic	Timd2-Havcr1
4	intergenic	Triml2-Zfp42
4	intergenic	Atp6v1h-Oprk1
4	intergenic	Lmo7-Kctd12/Mir5130
4	intergenic	Galr3-Ankrd54
3	intergenic	D3Ertd751e-2610316D01Rik
4	intron	Zswim6
4	intron	Mefv
4	intergenic	Fstl5-Rapgef2
4	intergenic	B230216N24Rik-Pam
4	intergenic	Gm32200-Dtl
4	intergenic	Plxdc2-4930515L03Rik
4	intron	Ptk2
4	intergenic	Cypt15-Cypt14
4	intergenic	Tenm3-Gm2516
3	intron	Camta1
4	intergenic	Erich3-Tnni3k
4	intron	Rasal2
4	exon	Nup214
4	intergenic	Ctps2-Grpr
4	intron	1700034P13Rik
4	intergenic	Arrb1-Tpbgl
4	intron	Adam3
4	intergenic	Nav3-9230102K24Rik
3	intergenic	Gm13582-Tank
4	intergenic	Mir6899-Klf7
4	intergenic	Stox2-Trappc11
4	intron	Fryl
4	intron	Tacr1
4	intron	Iqck
4	intergenic	9330178D15Rik-4930570G19Rik
4	intergenic	Anapc4-5033403H07Rik
4	intergenic	Hnf4g-1110015O18Rik
4	exon	Gfi1
4	intron	Hormad1
4	intergenic	Slc9a9-Chst2
4	intron	Arid1a
4	intron	Prdm1
4	intergenic	Gm1653-Gm40178
4	intron	Celf2
4	intron	Defb1
4	intergenic	4930563F08Rik-Auts2
4	intergenic	Edn1-Phactr1
4	intron	Etv6
4	intergenic	Gm36793-Gm36851
4	intron	Pde6g
4	intergenic	Macrod2-Kif16b
4	intron	Ppp2r3a
4	intron	Ldah
3	intergenic	Tmem161b-Ccnh
4	intergenic	Boc-Nepro
4	intergenic	Usp3-Car12
4	intergenic	Rbfox1-Tmem114
4	intron	Fars2
4	intergenic	Kbtbd12-Mgll
4	intergenic	Ttc6-Sstr1
4	intron	Fli1
4	intergenic	Xrcc2-Actr3b
4	intergenic	Olfr875-Olfr876
4	intergenic	Tusc1-Caap1
4	intergenic	8430430B14Rik-4933433H22Rik
4	intergenic	Psmd7-Gm39244
4	intron	Ush2a
4	intron	Slc44a5
4	intergenic	Gap43-4932412D23Rik

Table S5. All off-targets for sgRNA2 predicted with CRISPOR.

Number of mismatch	Position	Gene
3	intergenic	Dok5-1700028P15Rik
4	intergenic	Pcdh20-Gm5088
4	intron	Cacna1c
4	intergenic	Slc45a4-Gpr20
4	intron	Rrh
4	intergenic	Baiap2l1-Baiap2l1/Dmrt1i
4	intergenic	Slitrk1-Slitrk6
4	intergenic	Pabpc2-Yipf5
4	intergenic	1700120G07Rik-Nps
4	intergenic	Hspa13-Samsn1
4	intron	Gli3
4	intergenic	D030045P18Rik-Grik2
4	exon	Yipf5
4	intergenic	Trim72-Itgam
4	intron	Slco2a1
4	intergenic	Gm7931-Slit2
4	intergenic	Mettl21c-Gm8251
4	intron	Dab1
4	intron	Zfp423
4	intron	4930442J19Rik
4	intron	LOC100125594
4	intron	Gm14144
4	intergenic	Ppp1cb-Gm15614
4	intergenic	6330420H09Rik-Gm51502
4	intron	Cbll1
4	intron	Sag
4	intron	Kalrn
4	intergenic	Gm29461-Slco6d1
4	intergenic	Cttnbp2-Lsm8
4	intron	Sgo2a
4	intergenic	Ccn2-Enpp1
4	exon	Gm33104
4	intergenic	Phf8-Huwe1
4	exon	Cox14
4	intron	Adgrf5
4	exon	Dmpk
4	intron	Dscaml1
3	intergenic	Gm28979-Ephb1
4	intron	Nedd9
4	intergenic	Gm21054-Cntn6
4	intron	Itgb6
4	intron	Mitf
4	intergenic	Oxsm-B230110C06Rik
3	intergenic	Cyb5a-Fbxo15
4	intergenic	Ksr1-Wsb1
4	intron	Matn2
4	intron	Coq3
4	intergenic	Mir672-Nexmif
4	intergenic	Irf2bp2-A630001O12Rik
4	exon	Cadm4
4	intron	Atp10a
4	intergenic	Papss1-Dkk2
4	exon	Steap2
4	intron	Dpysl5
4	intergenic	Aass-4930554P06Rik
4	intron	Etv6

Table S6. All off-targets for sgRNA3 predicted with CRISPOR.

Number of mismatch	Position	Gene
4	intron	Tbx15
4	intron	Cacna2d3
4	intergenic	4930474G06Rik/Gm33948-4930474G06Rik
4	intron	Coro2b
4	intergenic	Pou1f1-Chmp2b
4	intergenic	Arpc5-Ncf2
4	intergenic	Hgf-Gm28710
4	intergenic	H2ab1-Tgif2lx2/Tgif2lx1
3	intron	Kcnip4
4	intergenic	Bpnt2-4930423M02Rik
4	intron	Aak1
4	intron	Zfp704
4	intron	Asic2
4	intergenic	Mtx2-Rps6-ps4
4	intergenic	Tango6-Has3
4	intergenic	Fnd3c2-Mir6382
4	intergenic	Unc5d-4933433F19Rik
4	intergenic	4933402C06Rik-A230077H06Rik
4	exon	Tuft1
4	intron	Sh3rf3
4	intergenic	Tmem64-Calb1
4	intron	Dipk2b
4	intergenic	Btbd1-Tm6sf1
4	intergenic	Grb10-Cobl
4	intron	Rtn4rl1
4	intron	Nim1k
4	intron	Nxph1
4	intron	Mief1
4	intron	Cfap44
4	intergenic	Olfr142-Olfr1271
4	intergenic	Olfr1271-Olfr1272
4	intergenic	Airn-Mas1
4	intergenic	Vapb-Stx16
4	intron	Osbpl3
4	intergenic	Scgb1b24-Scgb2b26
4	intergenic	Dhrs2-Dhrs4
4	intergenic	Gm30662-Akap11
4	intron	Atxn7
4	intron	Sema5b
4	intergenic	Slc25a24-Vav3
4	intergenic	Ghitm-Nrg3

Table S7 MIQE reporter for *Clcn3* gene expression in wild type and *Clcn5* mutant mice

Clcn3 gene expression in wild type and Clcn5 mutant mice	
**Experimental design**	
Definition of experimental and control groups	Confirming the *Clcn3* mRNA expression in wild type and mutant
Number within each group	2 wild type and 2 mutant mice
Assay carried out by the core	WFIRM core facility
**Sample**	
Description	Kidney samples
Volume /mass of sample processed	20ul
Processing procedure	Kidney was homogenized and processed with miRNeasy Mini Kit (Cat No. 217004, QIAGEN, Germantown, MD)
If frozen, how quickly	-80 degree
If fixed with what and how quickly	Kidney was put in liquid nitrogen and frozen -80 degree
Sample storage conditions and duration	-80 degree, 1-2 months
**Nucleic acid extraction**	
Procedure and instrumentation	miRNeasy Mini Kit (Cat No. 217004, QIAGEN, Germantown, MD) procedure used
Name of kit and details of any modifications	miRNeasy Mini Kit (Cat No. 217004, QIAGEN, Germantown, MD)
Contamination assessment (DNA or RNA)	No contamination observed
Nucleic acid quantification	Nano Drop
Instrument and method	Nano Drop 2000 Spectrophotometer
Purity (A_{260}/A_{280})	2.02 ratio
RNA integrity: method/instrument	Comparative CT (ΔΔCT) and QuantStudio™ 3
RIN/RQI or Cq of 3' and 5' transcripts	N/A
Inhibition testing (Cq dilutions, spike or other)	N/A
**Reverse transcription**	
Complete reaction conditions	Template RNA up to 2ug, 2ul gDNA wiped out buffer, incubated 2 min at 42°C, and then immediately placed on ice, total volume 14ul. Reverse-transcription master mix- Quantiscript Reverse Transcriptase -1ul, Quantiscript RT buffer 5x - 4ul, RT Primer mix-1ul, and Template RAN 14ul. Total reaction volume 20ul, mixed well and incubated at 42°C for 1 hour.
Amount of RNA and reaction volume	2ul RNA, 20ul volume
Priming oligonucleotide and concentration	QuantiTect Qiagen -1ul
Reverse transcriptase and concentration	QuantiTect Qiagen -1ul
Temperature and time	1hours 42 degree
Manufacture of reagents and catalogue numbers	Qiagen cat. No. 205314

Storage conditions of cDNA	-80 degree
**qPCR target information**	
Gene symbol	CLCN3
Sequence accession number	NM_007711.3
Amplicon length	108 bp
In silicon specificity screen	https://pga.mgh.harvard.edu/cgi-bin/primerbank/new_search2.cgi
Location of each primer by exon and intron	Both in Exon 1
What splice variants are targeted	transcript variant b
**qPCR oligonucleotides**	
Primer sequence	CLCN3-F: AGCTACAACAGCATAACCAGC<br>CLCN3-R: GTCCCCGTCTAACAAATTGTCAT
RTPrimerDB identification number	2599550a1
**Manufacturer of oligonucleotides**	Eurofins Genomics LLC
Purification method	Reversed-phase HPLC followed by anion exchange HPLC
RT-qPCR protocol	In this method RNA First transcribe in to cDNA by reverse transcriptase from total RNA and cDNA was used as the template for the RT-qPCR
Complete reaction condition	50 °C-2, 95 °C-10 min Hold, 95 °C -15 sec, 60 °C- 1 min PCR -40X, 95 °C-15 sec, 60 °C 1 min, 95 °C-1 sec Melt Curve
Reaction volume and amount of cDNA	20ul reaction volume and 2ul cDNA
Primer, Mg^{2+} and dNTP concentration	SYBR™ Green PCR Master Mix-2x cat. 4367659
Polymerase identity and concentration	SYBR™ Green PCR Master Mix-2x cat. 4367659
Buffer/kit identity and manufacturer	Applied Biosystems by Thermo Fisher Scientific, life technologies # 4367659
**Exact chemical composition of the buffer**	
SYBR™ Green PCR Master Mix<br>TaqMan™ Universal PCR Master Mix	Power SYBR Green PCR Master Mix cat. 4367659<br>The 2X mix contains SYBR™ Green 1 Dye, AmpliTaq Gold DNA Polymerase LD, dNTPs with dUTP/dTTP blend, Passive Reference 1, and optimized buffer components. Contains 1 × 5 mL, sufficient for 200 reactions.<br><br>TaqMan™ Universal PCR Master Mix cat. 4304437<br>Supplied at 2X concentration. The mix contains AmpliTaq Gold™ DNA Polymerase, Uracil-DNA Glycosylase, dNTPs with dUTP, Passive Reference 1 and optimized buffer components. This pack contains one 5 mL tube, sufficient for 200 reactions.

Manufacturer of plates/tubes and cat number	Applied Biosystems by life technologies cat. N8010568
Reaction setup (manual/robotic)	Manual
Manufacturer of qPCR instrument	Applied Biosystems by Thermo Fisher Scientific
**Results for NTCs**	
Justification of number and choice of reference genes	Three technical replicates and Gapdh
Description of normalization method	Internal control gene
Number and concordance of biological replicates	Triplicates
Repeatability (intraassay variation)	
Statistical methods for results significance	Not performed due to less sample groups
Software (source, version)	Not used due to less sample groups

Table S8 MIQE reporter for *Clcn4* gene expression in wild type and *Clcn5* mutant mice

**Clcn4 gene expression in wild type and Clcn5 mutant mice**	
Item to check	Item used for RT-qPCR
**Experimental design**	
Definition of experimental and control groups	Confirming the *Clcn4* mRNA expression in wild type and mutant
Number within each group	2 wild type and 2 mutant mice
Assay carried out by the core	WFIRM core facility
**Sample**	
Description	Kidney samples
Volume /mass of sample processed	20ul
Processing procedure	Kidney was homogenized and processed with miRNeasy Mini Kit (Cat No. 217004, QIAGEN, Germantown, MD)
If frozen, how quickly	-80 degree
If fixed with what and how quickly	Kidney was put in liquid nitrogen and frozen -80 degree
Sample storage conditions and duration	-80 degree, 1-2 months
**Nucleic acid extraction**	
Procedure and instrumentation	miRNeasy Mini Kit (Cat No. 217004, QIAGEN, Germantown, MD) procedure used
Name of kit and details of any modifications	miRNeasy Mini Kit (Cat No. 217004, QIAGEN, Germantown, MD)
Contamination assessment (DNA or RNA)	No contamination observed
Nucleic acid quantification	Nano Drop
Instrument and method	Nano Drop 2000 Spectrophotometer
Purity (A_{260}/A_{280})	2.0 ratio
RNA integrity: method/instrument	Comparative CT (ΔΔCT) and QuantStudio™ 3
RIN/RQI or Cq of 3’ and 5’ transcripts	N/A
Inhibition testing (Cq dilutions, spike or other)	N/A
**Reverse transcription**	
Complete reaction conditions	Template RNA up to 2ug, 2ul gDNA wiped out buffer, incubated 2 min at 42°C, and then immediately placed on ice, total volume 14ul. Reverse-transcription master mix- Quantiscript Reverse Transcriptase -1ul, Quantiscript RT buffer 5x - 4ul, RT Primer mix-1ul, and Template RAN 14ul. Total reaction volume 20ul, mixed well and incubated at 42°C for 1 hour.
Amount of RNA and reaction volume	2ul RNA, 20ul volume
Priming oligonucleotide and concentration	QuantiTect Qiagen -1ul
Reverse transcriptase and concentration	QuantiTect Qiagen -1ul
Temperature and time	1hours 42 degree
Manufacture of reagents and catalogue numbers	Qiagen cat. No. 205314

Storage conditions of cDNA	-80 degree
**qPCR target information**	
Gene symbol	CLCN4
Sequence accession number	NM_011334
Amplicon length	175 bp
In silicon specificity screen	https://pga.mgh.harvard.edu/cgi-bin/primerbank/new_search2.cgi
Location of each primer by exon and intron	Both in Exon 1
What splice variants are targeted	transcript variant a
**qPCR oligonucleotides**	
Primer sequence	CLCN4-F: CGGGATACCGACAGACATAGG<br>CLCN4-R: TGAGGTCCGTCATCCAATCCA
RTPrimerDB identification number	110625939c1
**Manufacturer of oligonucleotides**	Eurofins Genomics LLC
Purification method	Reversed-phase HPLC followed by anion exchange HPLC
RT-qPCR protocol	In this method RNA First transcribe in to cDNA by reverse transcriptase from total RNA and cDNA was used as the template for the RT-qPCR
Complete reaction condition	50 °C-2, 95 °C-10 min Hold, 95 °C -15 sec, 60 °C- 1 min PCR -40X, 95 °C-15 sec, 60 °C 1 min, 95 °C-1 sec Melt Curve
Reaction volume and amount of cDNA	20ul reaction volume and 2ul cDNA
Primer, Mg^{2+} and dNTP concentration	SYBR™ Green PCR Master Mix-2x cat. 4367659
Polymerase identity and concentration	SYBR™ Green PCR Master Mix-2x cat. 4367659
Buffer/kit identity and manufacturer	Applied Biosystems by Thermo Fisher Scientific, life technologies # 4367659
**Exact chemical composition of the buffer**	
SYBR™ Green PCR Master Mix<br>TaqMan™ Universal PCR Master Mix	Power SYBR Green PCR Master Mix cat. 4367659<br>The 2X mix contains SYBR™ Green 1 Dye, AmpliTaq Gold DNA Polymerase LD, dNTPs with dUTP/dTTP blend, Passive Reference 1, and optimized buffer components. Contains 1 × 5 mL, sufficient for 200 reactions.<br><br>TaqMan™ Universal PCR Master Mix cat. 4304437<br>Supplied at 2X concentration. The mix contains AmpliTaq Gold™ DNA Polymerase, Uracil-DNA Glycosylase, dNTPs with dUTP, Passive Reference 1 and optimized buffer components. This pack contains one 5 mL tube, sufficient for 200 reactions.

Manufacturer of plates/tubes and cat number	Applied Biosystems by life technologies cat. N8010568
Reaction setup (manual/robotic)	Manual
Manufacturer of qPCR instrument	Applied Biosystems by Thermo Fisher Scientific
**Results for NTCs**	
Justification of number and choice of reference genes	Three technical replicates and Gapdh
Description of normalization method	Internal control gene
Number and concordance of biological replicates	Triplicates
Repeatability (intraassay variation)	
Statistical methods for results significance	Not performed due to less sample groups
Software (source, version)	Not used due to less sample groups

Table S9 MIQE reporter for *CLCN5* gene expression in HEK293T cells with and without *CLCN5* LV transduction

***CLCN5* gene expression in HEK293T cells with and without *CLCN5* LV transduction**	
Item to check	Item used for RT-qPCR
**Experimental design**	
Definition of experimental and control groups	RT-qPCR confirmed LV vector mediated *CLCN5* mRNA expression in HEK293T cells. GFP-*CLCN5* expressing lentiviral vectors (10 ng p24) were transduced into 2.5x104 HEK293T cells.
Number within each group	3 in each group
Assay carried out by the core	WFIRM core facility
**Sample**	
Description	Transduced 293T cell samples
Volume /mass of sample processed	Cell pallet directly 350 RLT Plus Buffer
Processing procedure	Followed miRNeasy Mini Kit (Cat No. 217004, QIAGEN, Germantown, MD)
If frozen, how quickly	Not frozen
If fixed with what and how quickly	Not fixed
Sample storage conditions and duration	Not stored
**Nucleic acid extraction**	
Procedure and instrumentation	miRNeasy Mini Kit (Cat No. 217004, QIAGEN, Germantown, MD)
Name of kit and details of any modifications	miRNeasy Mini Kit (Cat No. 217004, QIAGEN, Germantown, MD)
Contamination assessment (DNA or RNA)	No contamination observed
Nucleic acid quantification	Nano Drop
Instrument and method	Nano Drop 2000 Spectrophotometer
Purity (A_{260}/A_{280})	2.02 ratio
RNA integrity: method/instrument	Comparative CT (ΔΔCT) and QuantStudio™ 3
RIN/RQI or Cq of 3' and 5' transcripts	N/A
Inhibition testing (Cq dilutions, spike or other)	N/A
**Reverse transcription**	
Complete reaction conditions	Template RNA up to 2ug, 2ul gDNA wiped out buffer, incubated 2 min at 42°C, and then immediately placed on ice, total volume 14ul. Reverse-transcription master mix- Quantiscript Reverse Transcriptase -1ul, Quantiscript RT buffer 5x - 4ul, RT Primer mix-1ul, and Template RAN 14ul. Total reaction volume 20ul, mixed well and incubated at 42°C for 1 hour.
Amount of RNA and reaction volume	2ul RNA, 20ul volume
Priming oligonucleotide and concentration	QuantiTect Qiagen -1ul
Reverse transcriptase and concentration	QuantiTect Qiagen -1ul

Temperature and time	1hours 42 degree
Manufacture of reagents and catalogue numbers	Qiagen cat. No. 205314
Storage conditions of cDNA	-80 degree
**qPCR target information**	
Gene symbol	*CLCN5*
Sequence accession number	NM_000084.5
Amplicon length	209 bp
In silicon specificity screen	Designed with Primer3 Input (Version 0.4.0)
Location of each primer by exon and intron	N/A
What splice variants are targeted	Codon optimized human *CLCN5* cDNA
**qPCR oligonucleotides**	
Primer sequence	hCLCN5-F-<br>TCTCGCCATGGATGTTATGA<br>hCLCN5-R- TCTTGCGTGCGTTTTCTATG
RTPrimerDB identification number	
**Manufacturer of oligonucleotides**	Eurofins Genomics LLC
Purification method	Reversed-phase HPLC followed by anion exchange HPLC
RT-qPCR protocol	In this method RNA First transcribe in to cDNA by reverse transcriptase from total RNA and cDNA was used as the template for the RT-qPCR
Complete reaction condition	50 °C-2, 95 °C-10 min Hold, 95 °C -15 sec, 60 °C- 1 min PCR -40X, 95 °C-15 sec, 60 °C 1 min, 95 °C-1 sec Melt Curve
Reaction volume and amount of cDNA	20ul reaction volume and 2ul cDNA
Primer, Mg^{2+} and dNTP concentration	SYBR™ Green PCR Master Mix-2x cat. 4367659
Polymerase identity and concentration	SYBR™ Green PCR Master Mix-2x cat. 4367659
Buffer/kit identity and manufacturer	Applied Biosystems by Thermo Fisher Scientific, life technologies # 4367659
**Exact chemical composition of the buffer**	
SYBR™ Green PCR Master Mix<br>TaqMan™ Universal PCR Master Mix	Power SYBR Green PCR Master Mix cat. 4367659<br>The 2X mix contains SYBR™ Green 1 Dye, AmpliTaq Gold DNA Polymerase LD, dNTPs with dUTP/dTTP blend, Passive Reference 1, and optimized buffer components. Contains 1 × 5 mL, sufficient for 200 reactions.<br><br>TaqMan™ Universal PCR Master Mix cat. 4304437<br>Supplied at 2X concentration. The mix contains AmpliTaq Gold™ DNA Polymerase, Uracil-DNA Glycosylase, dNTPs with dUTP, Passive Reference 1 and optimized buffer

	components. This pack contains one 5 mL tube, sufficient for 200 reactions.
Manufacturer of plates/tubes and cat number	Applied Biosystems by life technologies cat. N8010568
Reaction setup (manual/robotic)	Manual
Manufacturer of qPCR instrument	Applied Biosystems by Thermo Fisher Scientific
**Results for NTCs**	
Justification of number and choice of reference genes	Three technical replicates and Gapdh
Description of normalization method	Internal control gene
Number and concordance of biological replicates	Triplicates
Repeatability (intraassay variation)	N/A
Statistical methods for results significance	Not performed due to less sample groups
Software (source, version)	Not used due to less sample groups

Table S10 MIQE reporter for LV DNA in different organs following GFP LV delivery

Confirming the GFP LV delivery in injected mice of different organs	
Item to check	Item used for qPCR
**Experimental design**	
Definition of experimental and control groups	Confirming the GFP LV delivery in injected mice of different organs
Number within each group	2 wild type and 2 mutant mice
Assay carried out by the core	WFIRM core facility
**Sample**	
Description	Kidney, Bladder, Heart, Liver, Muscle, Spleen, Testis samples were collected and homogenized for DNA isolation
Volume /mass of sample processed	200ul
Processing procedure	Organs samples homogenized and processed DNeasy Blood & Tissue Kit (Qiagen) cat. No. 69504 and 69505
If frozen, how quickly	Kidney was put in liquid nitrogen and frozen -80 degree
Sample storage conditions and duration	-80 degree, 1-2 months
**Nucleic acid extraction**	
Procedure and instrumentation	DNeasy Blood & Tissue Kit (Qiagen) cat. No. 69504 and 69505
Name of kit and details of any modifications	DNeasy Blood & Tissue Kit (Qiagen) cat. No. 69504 and 69505
Contamination assessment (DNA or RNA)	No contamination observed
Nucleic acid quantification	Nano Drop
Instrument and method	Nano Drop 2000 Spectrophotometer
Purity (A_{260}/A_{280})	1.9 ratio
Method/instrument	Comparative CT (ΔΔCT) and QuantStudio™ 3
RIN/RQI or Cq of 3’ and 5’ transcripts	N/A
Inhibition testing (Cq dilutions, spike or other)	N/A
**qPCR target information**	
Gene symbol	EGFP
Sequence accession number	MW987528.1
Amplicon length	187 bp
In silicon specificity screen	Designed with Primer3 Input (Version 0.4.0)
Location of each primer by exon and intron	N/A
What splice variants are targeted	N/A
**qPCR oligonucleotides**	
Primer sequence	GFPF: acgtaaacggccacaagttc<br>GFPR: aagtcgtgctgcttcatgg
RTPrimerDB identification number	N/A
**Manufacturer of oligonucleotides**	Eurofins Genomics LLC
Purification method	Reversed-phase HPLC followed by anion exchange HPLC

qPCR protocol	In this method DNA isolated from different organs was used as the template for the qPCR
Complete reaction condition	50 °C-2, 95 °C-10 min Hold, 95 °C -15 sec, 60 °C- 1 min PCR -40X, 95 °C-15 sec, 60 °C 1 min, 95 °C-1 sec Melt Curve
Reaction volume and amount of DNA	20ul reaction volume and 2ul DNA
Primer, Mg^{2+} and dNTP concentration	SYBR™ Green PCR Master Mix-2x cat. 4367659
Polymerase identity and concentration	SYBR™ Green PCR Master Mix-2x cat. 4367659
Buffer/kit identity and manufacturer	Applied Biosystems by Thermo Fisher Scientific, life technologies # 4367659
**Exact chemical composition of the buffer**	
SYBR™ Green PCR Master Mix<br>TaqMan™ Universal PCR Master Mix	Power SYBR Green PCR Master Mix cat. 4367659<br>The 2X mix contains SYBR™ Green 1 Dye, AmpliTaq Gold DNA Polymerase LD, dNTPs with dUTP/dTTP blend, Passive Reference 1, and optimized buffer components. Contains 1 × 5 mL, sufficient for 200 reactions.<br><br>TaqMan™ Universal PCR Master Mix cat. 4304437<br>Supplied at 2X concentration. The mix contains AmpliTaq Gold™ DNA Polymerase, Uracil-DNA Glycosylase, dNTPs with dUTP, Passive Reference 1 and optimized buffer components. This pack contains one 5 mL tube, sufficient for 200 reactions.
Manufacturer of plates/tubes and cat number	Applied Biosystems by life technologies cat. N8010568
Reaction setup (manual/robotic)	Manual
Manufacturer of qPCR instrument	Applied Biosystems by Thermo Fisher Scientific
**Results for NTCs**	
Justification of number and choice of reference genes	Three technical replicates and Gapdh
Description of normalization method	Internal control gene
Number and concordance of biological replicates	Triplicates
Repeatability (intraassay variation)	
Statistical methods for results significance	Not performed due to less sample groups
Software (source, version)	Not used due to less sample groups

Table S11 MIQE reporter for LV DNA in the kidneys of mice following the first and second *CLCN5* LV delivery

Confirming the gDNA Psi signal for LV DNA in the kidneys of mice following the first and second *CLCN5* LV delivery	
Item to check	Item used for qPCR
**Experimental design**	
Definition of experimental and control groups	Confirming the gDNA Psi signal for LV DNA in the kidneys of mice following the first and second *CLCN5* LV delivery
Number within each group	3 in each group
Assay carried out by the core	WFIRM core facility
**Sample**	
Description	Kidney samples were collected and homogenized for DNA isolation
Volume /mass of sample processed	200ul
Processing procedure	Kidney samples homogenized and processed DNeasy Blood & Tissue Kit (Qiagen) cat. No. 69504 and 69505
If frozen, how quickly	Kidney was put in liquid nitrogen and frozen -80 degree
Sample storage conditions and duration	-80 degree, 1-2 months
**Nucleic acid extraction**	
Procedure and instrumentation	DNeasy Blood & Tissue Kit (Qiagen) cat. No. 69504 and 69505 procedure used
Name of kit and details of any modifications	DNeasy Blood & Tissue Kit (Qiagen) cat. No. 69504 and 69505
Contamination assessment (DNA or RNA)	No contamination observed
Nucleic acid quantification	Nano Drop
Instrument and method	Nano Drop 2000 Spectrophotometer
Purity (A_{260}/A_{280})	1.9 ratio
Method/instrument	Comparative CT (ΔΔCT) and QuantStudio™ 3
RIN/RQI or Cq of 3' and 5' transcripts	N/A
Inhibition testing (Cq dilutions, spike or other)	N/A
**qPCR target information**	
Gene symbol	A region near HIV-1 LTR/gag
Sequence accession number	K03455.1
Amplicon length	127 bp
In silicon specificity screen	Based on Nat. Protoc., 3 (7) (2008), pp. 1240-1248
Location of each primer by exon and intron	N/A
What splice variants are targeted	Human immunodeficiency virus type 1 (HXB2)
**qPCR oligonucleotides**	
Primer sequence	Psi-F: TCTCGACGCAGGACTCG<br>Psi-R: TACTGACGCTCTCGCACC
RTPrimerDB identification number	N/A

**Manufacturer of oligonucleotides**	Eurofins Genomics LLC
Purification method	Reversed-phase HPLC followed by anion exchange HPLC
qPCR protocol	In this method DNA isolated from different organs was used as the template for the qPCR
Complete reaction condition	50 °C-2, 95 °C-10 min Hold, 95 °C -15 sec, 60 °C- 1 min PCR -40X, 95 °C-15 sec, 60 °C 1 min, 95 °C-1 sec Melt Curve
Reaction volume and amount of DNA	20ul reaction volume and 2ul DNA
Primer, Mg^{2+} and dNTP concentration	SYBR™ Green PCR Master Mix-2x cat. 4367659
Polymerase identity and concentration	SYBR™ Green PCR Master Mix-2x cat. 4367659
Buffer/kit identity and manufacturer	Applied Biosystems by Thermo Fisher Scientific, life technologies # 4367659
**Exact chemical composition of the buffer**	
SYBR™ Green PCR Master Mix<br>TaqMan™ Universal PCR Master Mix	Power SYBR Green PCR Master Mix cat. 4367659<br>The 2X mix contains SYBR™ Green 1 Dye, AmpliTaq Gold DNA Polymerase LD, dNTPs with dUTP/dTTP blend, Passive Reference 1, and optimized buffer components. Contains 1 × 5 mL, sufficient for 200 reactions.<br><br>TaqMan™ Universal PCR Master Mix cat. 4304437<br>Supplied at 2X concentration. The mix contains AmpliTaq Gold™ DNA Polymerase, Uracil-DNA Glycosylase, dNTPs with dUTP, Passive Reference 1 and optimized buffer components. This pack contains one 5 mL tube, sufficient for 200 reactions.
Manufacturer of plates/tubes and cat number	Applied Biosystems by life technologies cat. N8010568
Reaction setup (manual/robotic)	Manual
Manufacturer of qPCR instrument	Applied Biosystems by Thermo Fisher Scientific
**Results for NTCs**	
Justification of number and choice of reference genes	Three technical replicates and Gapdh
Description of normalization method	Internal control gene
Number and concordance of biological replicates	Triplicates
Repeatability (intraassay variation)	
Statistical methods for results significance	Not performed due to less sample groups
Software (source, version)	Not used due to less sample groups

Table S12 MIQE reporter for *CLCN5* expression in the kidneys of mice following the first and second *CLCN5* LV delivery

***CLCN5* expression in the kidneys of mice following the first and second *CLCN5* LV delivery**	
Item to check	Item used for RT-qPCR
**Experimental design**	
Definition of experimental and control groups	Confirming the *Clcn5* mRNA expression in injected and not injected CLCN5 LV mice.
Number within each group	2 wild type and 2 mutant mice
Assay carried out by the core	WFIRM core facility
**Sample**	
Description	Kidney samples
Volume /mass of sample processed	200ul
Processing procedure	Kidney was homogenized and processed with miRNeasy Mini Kit (Cat No. 217004, QIAGEN, Germantown, MD)
If frozen, how quickly	-80 degree
If fixed with what and how quickly	Kidney was put in liquid nitrogen and frozen -80 degree
Sample storage conditions and duration	-80 degree, 1-2 months
**Nucleic acid extraction**	
Procedure and instrumentation	miRNeasy Mini Kit (Cat No. 217004, QIAGEN, Germantown, MD) procedure used
Name of kit and details of any modifications	miRNeasy Mini Kit (Cat No. 217004, QIAGEN, Germantown, MD)
Contamination assessment (DNA or RNA)	No contamination observed
Nucleic acid quantification	Nano Drop
Instrument and method	Nano Drop 2000 Spectrophotometer
Purity (A_{260}/A_{280})	2.0 ratio
RNA integrity: method/instrument	Comparative CT (ΔΔCT) and QuantStudio™ 3
RIN/RQI or Cq of 3' and 5' transcripts	N/A
Inhibition testing (Cq dilutions, spike or other)	N/A
**Reverse transcription**	
Complete reaction conditions	Template RNA up to 2ug, 2ul gDNA wiped out buffer, incubated 2 min at 42°C, and then immediately placed on ice, total volume 14ul. Reverse-transcription master mix- Quantiscript Reverse Transcriptase -1ul, Quantiscript RT buffer 5x - 4ul, RT Primer mix-1ul, and Template RAN 14ul. Total reaction volume 20ul, mixed well and incubated at 42°C for 1 hour.
Amount of RNA and reaction volume	2ul RNA, 20ul volume
Priming oligonucleotide and concentration	QuantiTect Qiagen -1ul
Reverse transcriptase and concentration	QuantiTect Qiagen -1ul
Temperature and time	1hours 42 degree

Manufacture of reagents and catalogue numbers	Qiagen cat. No. 205314
Storage conditions of cDNA	-80 degree
**qPCR target information**	
Gene symbol	CLCN5
Sequence accession number	NM_000084.5
Amplicon length	209 bp
In silicon specificity screen	Designed with Primer3 Input (Version 0.4.0)
Location of each primer by exon and intron	N/A
What splice variants are targeted	Codon optimized human *CLCN5* cDNA
**qPCR oligonucleotides**	
Primer sequence	hCLCN5-F(cDNA)- TCTCGCCATGGATGTTATGA hCLCN5-R(cDNA)- TCTTGCGTGCGTTTTCTATG
RTPrimerDB identification number	N/A
**Manufacturer of oligonucleotides**	Eurofins Genomics LLC
Purification method	Reversed-phase HPLC followed by anion exchange HPLC
RT-qPCR protocol	In this method RNA First transcribe in to cDNA by reverse transcriptase from total RNA and cDNA was used as the template for the RT-qPCR
Complete reaction condition	50 °C-2, 95 °C-10 min Hold, 95 °C -15 sec, 60 °C- 1 min PCR -40X, 95 °C-15 sec, 60 °C 1 min, 95 °C-1 sec Melt Curve
Reaction volume and amount of cDNA	20ul reaction volume and 2ul cDNA
Primer, Mg^{2+} and dNTP concentration	SYBR™ Green PCR Master Mix-2x cat. 4367659
Polymerase identity and concentration	SYBR™ Green PCR Master Mix-2x cat. 4367659
Buffer/kit identity and manufacturer	Applied Biosystems by Thermo Fisher Scientific, life technologies # 4367659
**Exact chemical composition of the buffer**	
SYBR™ Green PCR Master Mix<br>TaqMan™ Universal PCR Master Mix	Power SYBR Green PCR Master Mix cat. 4367659<br>The 2X mix contains SYBR™ Green 1 Dye, AmpliTaq Gold DNA Polymerase LD, dNTPs with dUTP/dTTP blend, Passive Reference 1, and optimized buffer components. Contains 1 × 5 mL, sufficient for 200 reactions.<br><br>TaqMan™ Universal PCR Master Mix cat. 4304437<br>Supplied at 2X concentration. The mix contains AmpliTaq Gold™ DNA Polymerase, Uracil-DNA Glycosylase, dNTPs with dUTP, Passive Reference 1 and optimized buffer components. This pack contains one 5 mL tube, sufficient for 200 reactions.

Manufacturer of plates/tubes and cat number	Applied Biosystems by life technologies cat. N8010568
Reaction setup (manual/robotic)	Manual
Manufacturer of qPCR instrument	Applied Biosystems by Thermo Fisher Scientific
**Results for NTCs**	
Justification of number and choice of reference genes	Three technical replicates and Gapdh
Description of normalization method	Internal control gene
Number and concordance of biological replicates	Triplicates
Repeatability (intraassay variation)	N/A
Statistical methods for results significance	Not performed due to less sample groups
Software (source, version)	Not used due to less sample groups

Table S13. Sequence information for codon-optimized human CLCN5 cDNA

Name	Sequence
Codon optimized human *CLCN5* cDNA	ctcgagccaccATGGATTTCCTgGAGGAACCAATACCAGGTGTAGGAACATATGACGATTT<br>CAATACTATAGACTGGGTGCGAGAGAAATCACGCGATCGAGACAGACACCGGGAGA<br>TCACGAATAAGTCTAAGGAATCTACCTGGGCCCTCATTCACAGTGTGTCAGACGCTT<br>TTAGCGGATGGCTGCTTATGCTTCTGATTGGACTTCTTAGTGGTAGTTTGGCGGGCCT<br>GATAGACATTAGCGCGCACTGGATGACTGATCTTAAAGAAGGCATATGCACGGGGG<br>GATTTTGGTTCAACCACGAACATTGCTGCTGGAACTCCGAGCATGTGACATTCGAGG<br>AGAGGGACAAGTGCCCCGAGTGGAATAGTTGGAGCCAACTGATAATTTCTACAGAT<br>GAGGGGGCTTTTGCCTATATAGTTAATTATTTCATGTATGTTTTGTGGGCCCTCCTCTT<br>CGCCTTCCTCGCGGTATCCCTCGTTAAGGTCTTTGCCCCATATGCCTGTGGCTCTGGT<br>ATTCCAGAAATAAAAACTATCCTTTCTGGATTTATAATCAGGGGATATCTGGGCAAG<br>TGGACGTTGGTCATTAAGACAATCACCCTTGTCCTTGCTGTATCTTCAGGGTTGTCCT<br>TGGGCAAAGAGGGTCCTCTCGTTCACGTAGCTTGCTGCTGTGGGAACATCCTTTGCC<br>ATTGTTTCAATAAATATAGGAAGAACGAAGCAAAGCGCCGAGAAGTTCTGAGCGCA<br>GCAGCGGCCGCAGGTGTCAGTGTTGCCTTCGGGGCTCCTATAGGAGGGGTACTGTTT<br>AGTCTCGAAGAAGTGTCATATTACTTTCCTCTCAAGACACTGTGGAGGTCCTTTTTTG<br>CAGCCCTGGTCGCGGCTTTTACTCTGCGCTCTATTAATCCTTTTGGAAACAGCAGACT<br>TGTGCTGTTCTACGTCGAATTCCACACCCCGTGGCATTTGTTTGAACTCGTACCCTTT<br>ATTTTGCTGGGGATTTTCGGTGGATTGTGGGGTGCTCTGTTCATACGCACTAACATTG<br>CGTGGTGCCGGAAGAGGAAGACTACTCAGTTGGGCAAATACCCAGTTATTGAGGTCC<br>TCGTCGTTACAGCTATCACAGCAATTCTTGCGTTCCCCAACGAGTACACACGGATGTC<br>TACATCCGAACTGATTAGCGAACTGTTCAATGATTGTGGGCTCTTGGACTCCTCAAA<br>ACTGTGCGATTATGAAAATCGATTTAATACATCAAAGGGCGGAGAACTTCCCGATCG<br>GCCGGCTGGAGTGGGAGTATACTCCGCTATGTGGCAGCTGGCGTTGACGCTCATACT<br>CAAAATCGTCATTACCATATTCACTTTTGGAATGAAGATTCCCTCAGGTCTCTTTATC<br>CCTAGTATGGCAGTTGGTGCGATTGCGGGACGGCTCCTGGGCGTTGGCATGGAGCAG<br>CTGGCTTATTACCATCAGGAGTGGACCGTATTCAATAGCTGGTGCTCTCAGGGCGCT<br>GATTGCATCACACCAGGCCTGTATGCCATGGTAGGCGCTGCTGCTTGTCTTGGAGGG<br>GTGACTAGGATGACGGTTTCTCTCGTCGTGATAATGTTCGAGCTTACTGGGGGTCTTG<br>AGTACATTGTGCCCCTGATGGCGGCGGCAATGACATCCAAATGGGTGGCGGATGCGT<br>TGGGTAGGGAAGGGATATACGATGCACATATTCGCCTTAATGGCTACCCATTTTTGG<br>AGGCTAAGGAAGAATTTGCACATAAAACTCTCGCCATGGATGTTATGAAACCGAGAC<br>GAAACGACCCATTGCTTACAGTACTTACACAGGATTCCATGACCGTTGAGGACGTGG<br>AAACAATAATATCTGAAACAACTTATAGTGGCTTTCCCGTCGTCGTATCCCGAGAAT<br>CACAAAGGTTGGTAGGATTCGTGCTGCGACGCGACCTGATCATATCCATAGAAAACG<br>CACGCAAGAAGCAAGACGGGGTAGTGTCCACGTCTATAATTTATTTCACCGAGCATA<br>GCCCTCCCTTGCCTCCATATACTCCGCCTACACTGAAACTTCGAAACATCCTCGATTT<br>GTCTCCTTTTACAGTAACCGACCTTACTCCAATGGAAATCGTAGTAGACATATTTAGA<br>AAGCTTGGATTGAGGCAATGCCTGGTTACCCACAACGGTCGGTTGCTCGGATAATA

	ACGAAGAAGGACGTACTCAAACATATAGCACAAATGGCAAACCAGGACCCgGATTC AATCTTGTTCAACTAGtctaga
Human CLCN5 Protein	MDFLEEPIPGVGTYDDFNTIDWVREKSRDRDRHREITNKSKESTWALIHSVSDAFSGWLL MLLIGLLSGSLAGLIDISAHWMTDLKEGICTGGFWFNHEHCCWNSEHVTFEERDKCPEW NSWSQLIISTDEGAFAYIVNYFMYVLWALLFAFLAVSLVKVFAPYACGSGIPEIKTILSGFI IRGYLGKWTLVIKTITLVLAVSSGLSLGKEGPLVHVACCCGNILCHCFNKYRKNEAKRRE VLSAAAAAGVSVAFGAPIGGVLFSLEEVSYYFPLKTLWRSFFAALVAAFTLRSINPFGNS RLVLFYVEFHTPWHLFELVPFILLGIFGGLWGALFIRTNIAWCRKRKTTQLGKYPVIEVLV VTAITAILAFPNEYTRMSTSELISELFNDCGLLDSSKLCDYENRFNTSKGGELPDRPAGVG VYSAMWQLALTLILKIVITIFTFGMKIPSGLFIPSMAVGAIAGRLLGVGMEQLAYYHQEW TVFNSWCSQGADCITPGLYAMVGAAACLGGVTRMTVSLVVIMFELTGGLEYIVPLMAA AMTSKWVADALGREGIYDAHIRLNGYPFLEAKEEFAHKTLAMDVMKPRRNDPLLTVLT QDSMTVEDVETIISETTYSGFPVVVSRESQRLVGFVLRRDLIISIENARKKQDGVVSTSIIYF TEHSPPLPPYTPPTLKLRNILDLSPFTVTDLTPMEIVVDIFRKLGLRQCLVTHNGRLLGIITK KDVLKHIAQMANQDPDSILFN*

Table S14. Sequence information for primers

Name	Sequence	Purpose
hCLCN5-F(cDNA)	TCTCGCCATGGATGTTATGA	RT-qPCR to detect transgene *hCLCN5* expression.
hCLCN5-R(cDNA)	TCTTGCGTGCGTTTTCTATG	
mCLCN5-EF:	CCCTGGTGTAGGGACCTATG	RT-PCR to detect endogenous mouse *Clcn5* expression.
mCLCN5-ER	CAGAATTCCAGCAACAGTGC	
CLCN5-KF2	AAGGGACAGTCATGGTCTGG	To amplify a 1000 bp DNA prodcut from *Clcn5* knockout mice but not wild type mice. For genotyping.
CLCN5-KR2	CAATGGCCTGTTGTGCATAC	
CLCN5-W2	CTGGGTTTCATGCATTTGTG	To amplify a 540 bp prodcut from wild type mice with primer CLCN5-KF2. For genotyping.
Psi-F	TCTCGACGCAGGACTCG	For detecting lentiviral genomic DNA integration.
Psi-R	TACTGACGCTCTCGCACC	
Itgb6-F	TCAGTTGCATGGATGGAGAG	To amplify predicted off-target of sgRNA 1 in Itgb6 gene.
Itgb6-R	AAGCTGAACTTTGCCCTTAGC	
CLCN5-off-1-1F	AAACACACCTCACTGGCAAAG	To amplify predicted off-target of sgRNA 1 7264 bp away from Mturn.
CLCN5-off-1-1R	GAATTCTCTCCAGGCTGCTG	
CLCN5-off-1-2F	GTACGCCAGTGTTGATGTGG	To amplify predicted off-target of sgRNA 1 40747 bp away from Camkmt.
CLCN5-off-1-2R	TGCGACACCAGTCAGATAGC	
CLCN5-off-1-3F	CTCAAATGCAATTCCCTTCC	To amplify predicted off-target of sgRNA 1 in a intergenic region on chromosome 3.
CLCN5-off-1-3R	CCACTGGCATGCACATCTAC	
CLCN5-off-2-1F	CAAAGATCCTCTGCCTCTGC	To amplify predicted off-target of sgRNA 2 in a intergenic region on chromosome 2.
CLCN5-off-2-1R	TCTGGCCTTCATTTTTACCG	
CLCN5-off-2-2F	TTAATTCCCAATGCGGTAGC	To amplify predicted off-target of sgRNA 2 in a intergenic region on chromosome 3.
CLCN5-off-2-2R	ATTCAAGAGGTGGGTTCACG	
CLCN5-off-2-3F	TGATCTGCAGGGAACAGTTG	To amplify predicted off-target of sgRNA 2 in the intron of Cacna1c on chromosome 6.

CLCN5-off-2-3R	GCCTCTGGTACCTTG CTCAG	
CLCN5-off-3-1F	ATGGCTCCAAGAATC CAGTG	To amplify predicted off-target of sgRNA 3 in an intergenic region 1686 bp from Pou1f1 gene.
CLCN5-off-3-1R	ACCCCTCCTGATTTCT GTGC	
CLCN5-off-3-2F	GGACCCTCCCACATC CTAAC	To amplify predicted off-target of sgRNA 3 in an intergenic region 2409 bp from Fnd3c2 gene.
CLCN5-off-3-2R:	GTCCCACTTCTGAAG CAAGC	
CLCN5-off-3-3F	TCACTGCCAGGTAAG TGTGG	To amplify predicted off-target of sgRNA 3 in the intron of Coro2b gene.
CLCN5-off-3-3R	CTTGTCCAGCACATG GTGTC	
GFPF	ACGTAAACGGCCACA AGTTC	To detect GFP DNA.
GFPR	AAGTCGTGCTGCTTC ATGG	

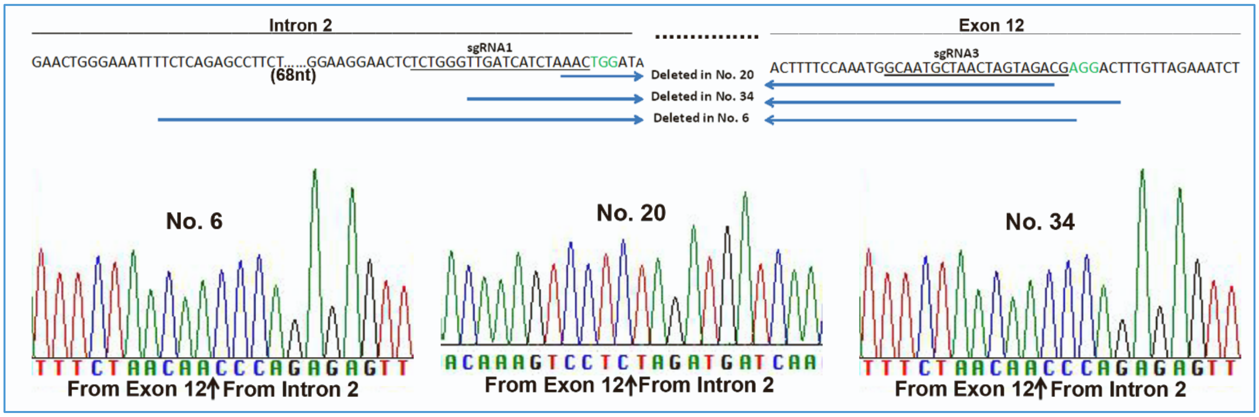

Figure S1. Confirming *Clcn5* gene knockout by DNA sequencing. Sequences above the horizontal arrows were deleted for the three founder females (No. 6, 20 and 34). The sgRNA target sequences (underlined) in intron 2 and exon 12 are shown. PAMs are in green. A reverse primer matching exon 12 was used for seqencing. The junctions bewtween intron 2 and exon 12 are indicated by a vertical arrow.

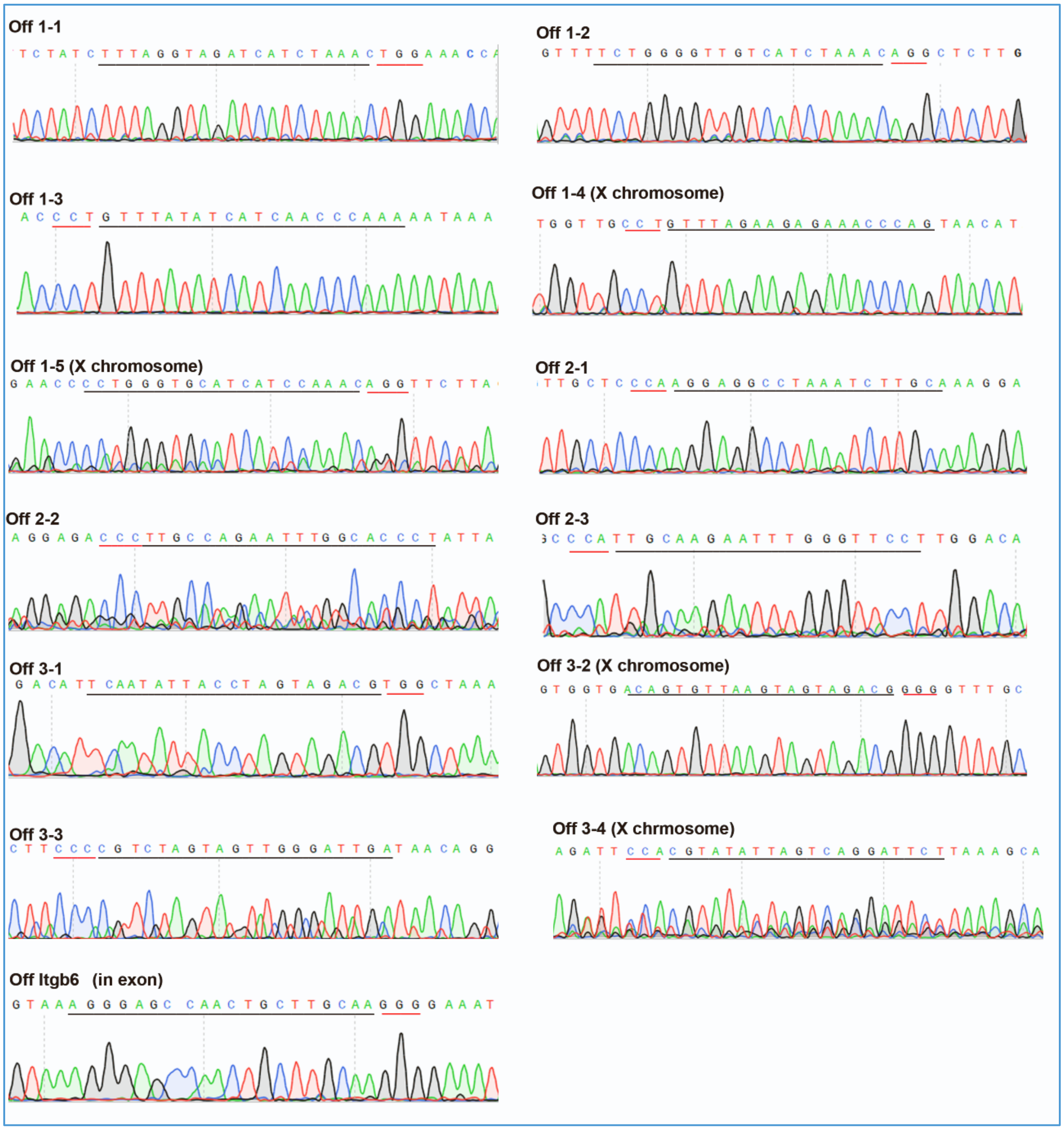

Figure S2. DNA sequencing analysis of predicted off-tatgets in *Clcn5* gene knockout mice. The protospacer adjacent motifs (or the reverse completemtary seqeunces) were underlined with red lines and the target sequences were underlined with black lines. Off 1, Off 2 and Off 3 were off-targets for sgRNA 1, sgRNA 2 and sgRNA 3 respectively. The last image was the only off-taregt on protein coding gene. The four off-targets on X chromosome were also labeled.

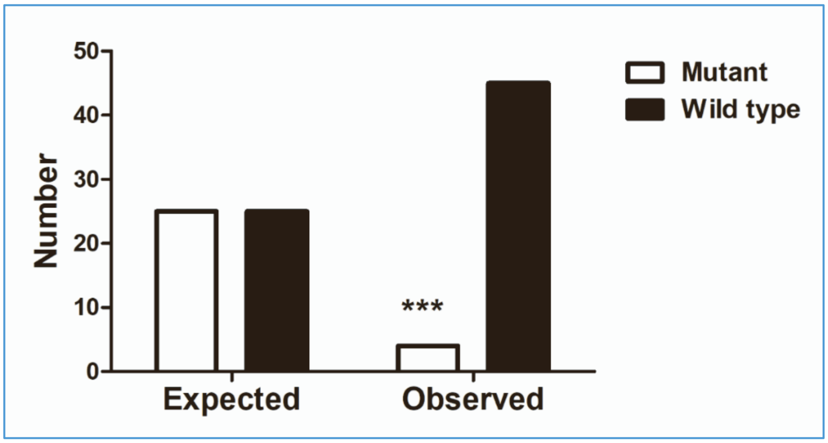

Figure S3. *Clcn5* mutant males were obtained less than expected. *** indicates p<0.001 in Fisher's exact test.

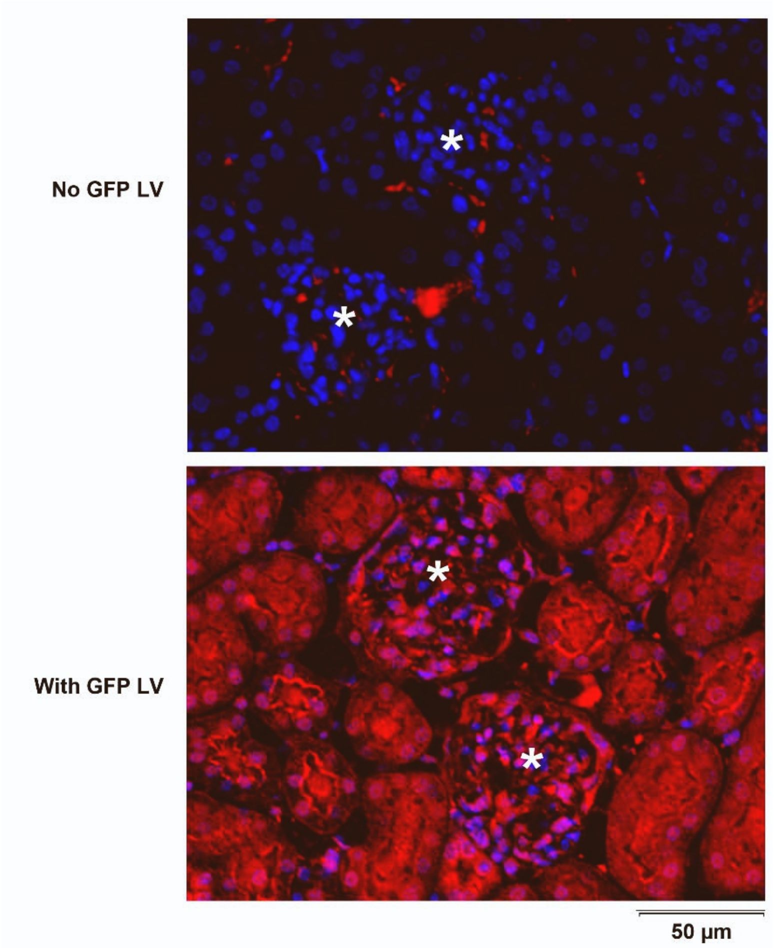

Figure S4. Observation of GFP expression in the glomeruli. Top iamge: Failure to observe GFP immunofluorescent signals in the kidney without GFP LV injection. Bottom image: Oberservation of GFP immunofluorescent signals in the tubular structures and the glomeruli of the kindeys with GFP LV injection. The glomeruli were marked by *.

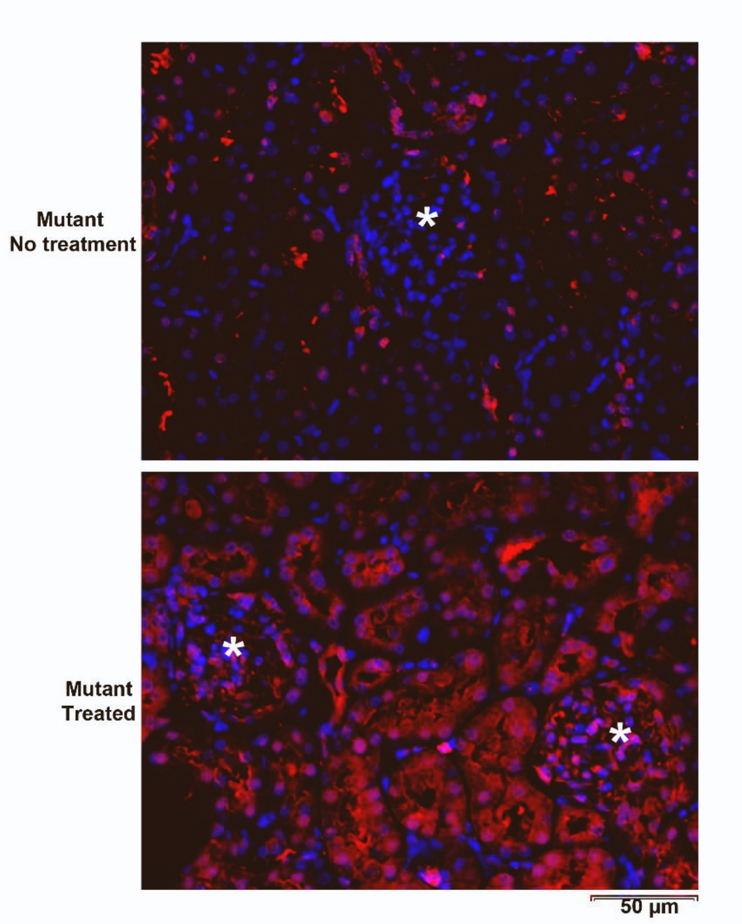

Figure S5. Observation of CLCN5 expression in the glomeruli of mutant mice. Top iamge: Failure to observe CLCN5 immunofluorescent signals in the kidney without CLCN5 LV injection. Bottom image: Oberservation of CLCN5 immunofluorescent signals in the tubular structures and the glomeruli of the kindeys with CLCN5 LV injection. The glomeruli were marked by *.

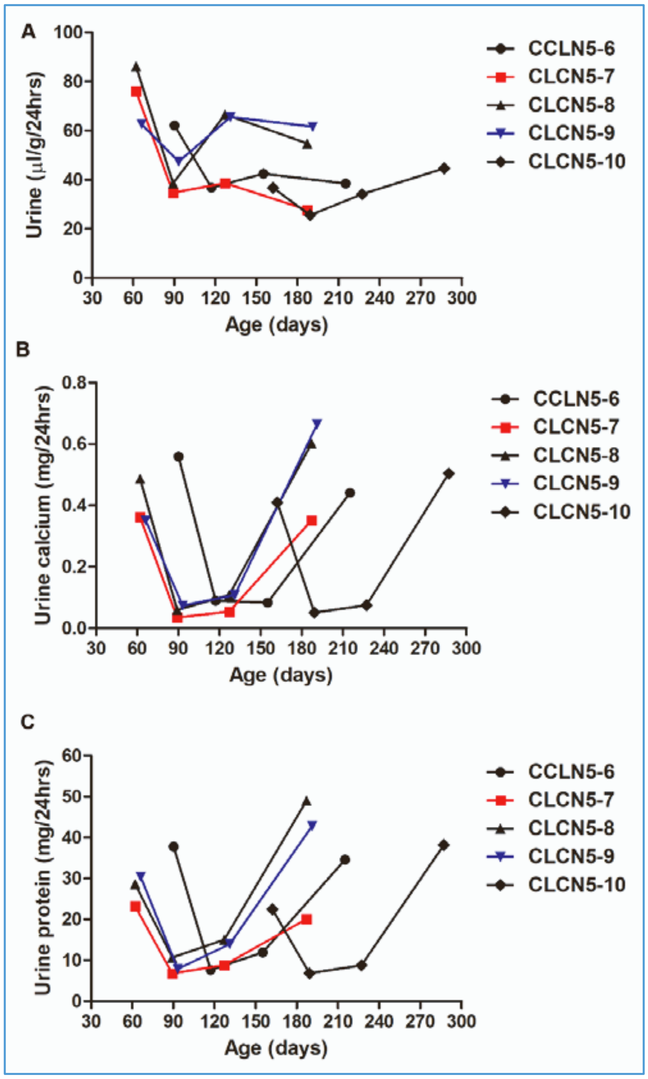

Figure S6. Effects of delivering CLCN5 LV to the left kidney. **A**. Urine volume. **B**. Urine calcium. **C**. Urine protein. CLCN5 LV (280 ng p24) injection was performed on the day of the first datum point for each mouse. The urine was collected 37 days before LV injection. The second, third and fourth data points showed the actual time when the urine samples were collected.

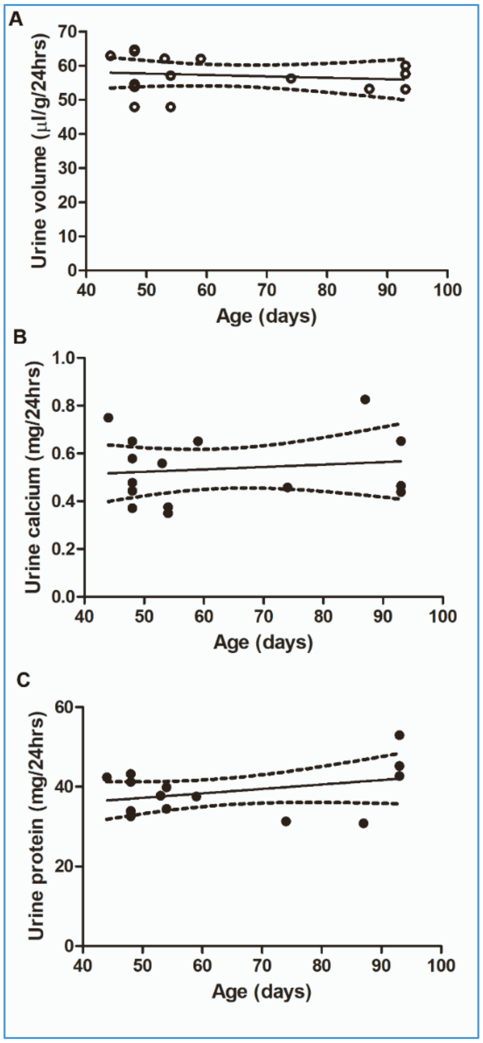

Figure S7. Age did not greatly affect the urine parameters of mutant mice. **A**. Urine volume. **B**. Urine calcium. **C**. Urine protein. Each datum point was from a different male mutant mouse. The dashed lines show the 95% confidence intervals.

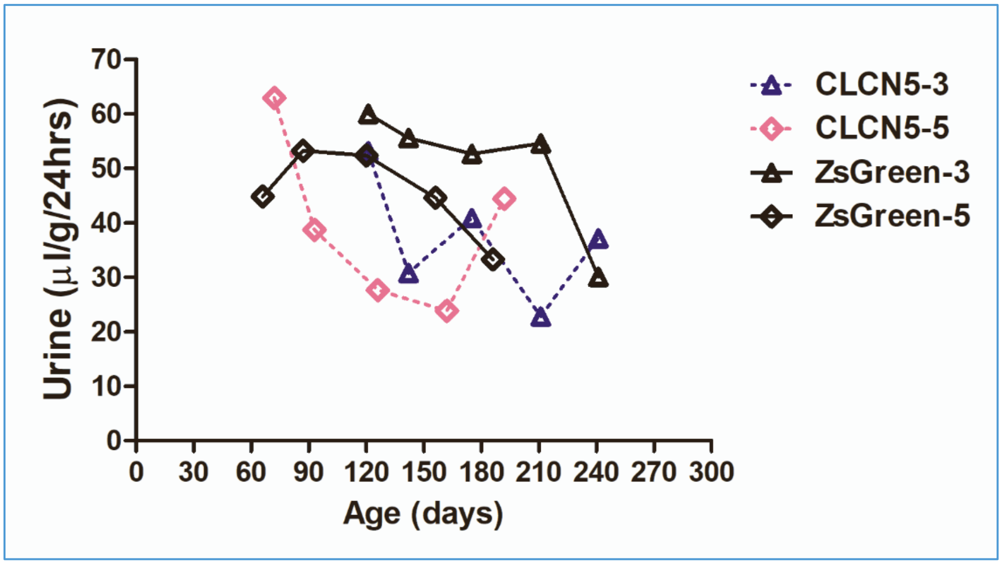

Figure S8. CLCN5 gene therapy on diuresis. Two of the 5 age-matched pairs were presented here for visibility. The other three pairs were shown in **Fig.7A**. Both kidneys were treated.

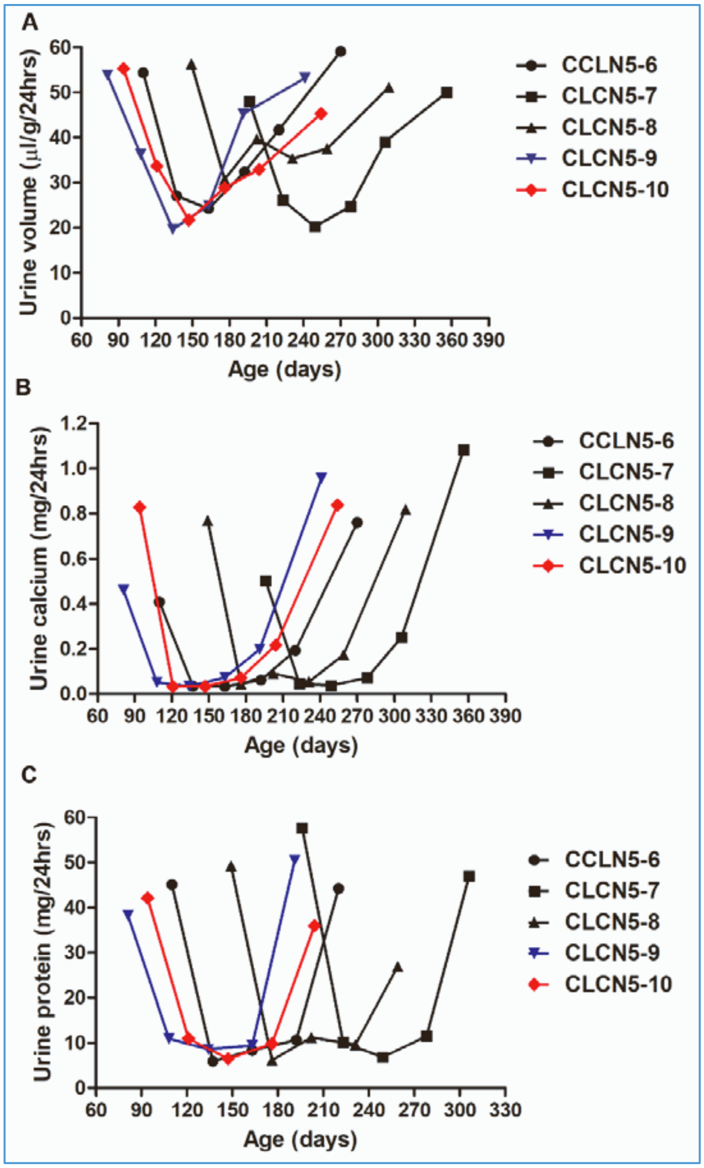

Figure S9. Effects of delivering CLCN5 LV to both kidneys. **A**. Urine volume. **B**. Urine calcium. **C**. Urine protein. CLCN5 LV (280 ng p24/kidney) injection was performed on the day of the first datum point for each mouse. The urine was collected 7 days before LV injection. The second, third, fourth and fifth data points showed the actual age when the urine samples were collected.

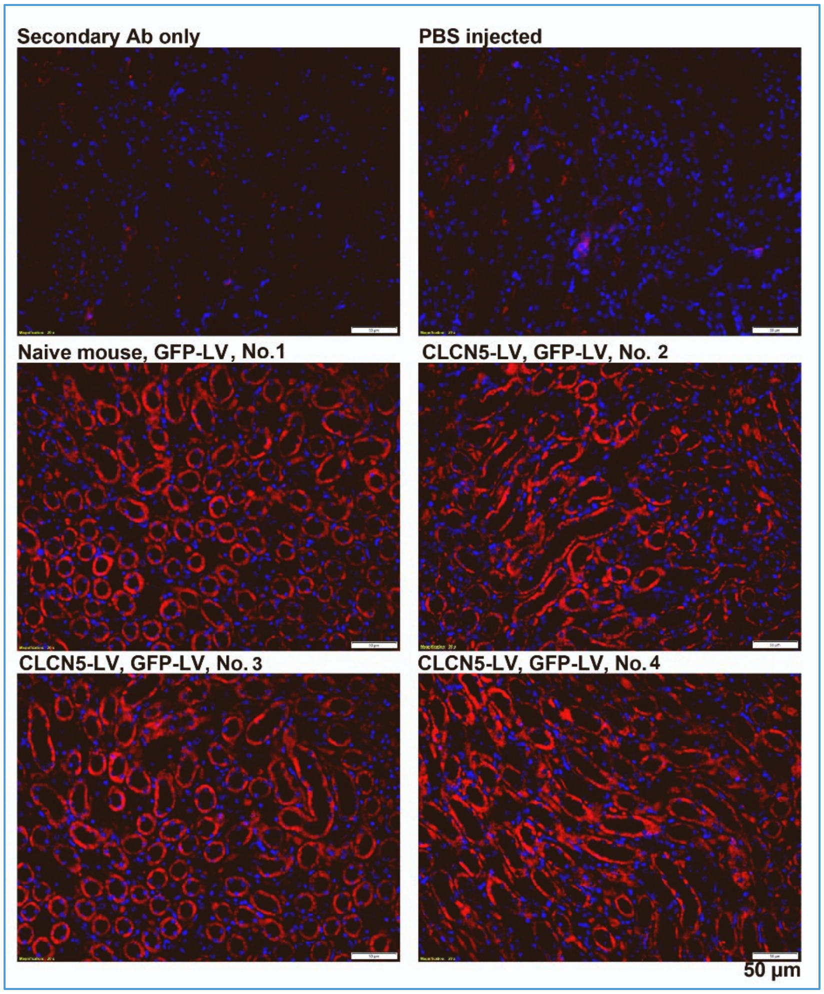

Figure S10. Detecting GFP protein by immunofluorescence in mouse kidney with and without CLCN5 LV injection. Naïve mouse was a 6-month wild type mouse receiving GFP LV injection without CLCN5 LV pre-injection. The GFP expression image for this mouse is a re-use of part of the image in Fig.3D (GFP-LV Injection Primary and Secondary Ab) since these images showed the GFP expression in the same mouse. The other three mice (CLCN5-LV, GFP-LV, No.2~4) were mutant mice that received GFP LV injection 10 months following CLCN5 LV injection. The mice were euthanized 2 weeks after GFP LV injection.
